# Ovarian Tumor Domain-Containing Proteases-Deubiquitylation Enzyme Gene *SsCI33130* Involved in the Regulation of Mating/Filamentation and Pathogenicity in *Sporisorium scitamineum*

**DOI:** 10.3389/fmicb.2021.746550

**Published:** 2021-10-05

**Authors:** Huizhong Li, Yichang Cai, Quanqing Deng, Han Bao, Jianwen Chen, Wankuan Shen

**Affiliations:** ^1^College of Agriculture, South China Agricultural University, Guangzhou, China; ^2^Sugarcane Research Laboratory, South China Agricultural University, Guangzhou, China; ^3^Scientific Observing and Experimental Station of Crop Cultivation in South China, Ministry of Agriculture and Rural Affairs, Guangzhou, China

**Keywords:** *Sporisorium scitamineum*, gene *SsCI33130*, OTU1-deubiquitylation enzyme, sexual mating, pathogenicity

## Abstract

Sugarcane is an important sugar crop. Sugarcane smut, caused by *Sporisorium scitamineum*, is a worldwide sugarcane disease with serious economic losses and lack of effective control measures. Revealing the molecular pathogenesis of *S. scitamineum* is very helpful to the development of effective prevention and control technology. Deubiquitinase removes ubiquitin molecules from their binding substrates and participates in a variety of physiological activities in eukaryotes. Based on the transcriptome sequencing data of two isolates (*Ss16* and *Ss47*) of *S. scitamineum* with different pathogenicities, *SsCI33130*, a gene encoding an OTU1-deubiquitin enzyme, was identified. The positive knockout mutants and complementary mutants of the *SsCI33130* gene were successfully obtained through polyethylene glycol-mediated protoplast transformation technology. In order to study the possible function of this gene in pathogenicity, phenotypic comparison of the growth, morphology, abiotic stress, sexual mating, pathogenicity, and gene expression levels of the knockout mutants, complementary mutants, and their wild type strains were conducted. The results demonstrated that the gene had almost no effect on abiotic stress, cell wall integrity, growth, and morphology, but was related to the sexual mating and pathogenicity of *S. scitamineum*. The sexual mating ability and pathogenicity between the knockout mutants or between the knockout mutant and wild type were more significantly reduced than between the wild types, the complementary mutants, or the wild types and complementary mutants. The sexual mating between the knockout mutants or between the knockout mutant and wild type could be restored by the exogenous addition of small-molecule signaling substances such as 5 mM cyclic adenosine monophosphate (cAMP) or 0.02 mM tryptophol. In addition, during sexual mating, the expression levels of tryptophol and cAMP synthesis-related genes in the knockout mutant combinations were significantly lower than those in the wild type combinations, while the expression levels in the complementary mutant combinations were restored to the level of the wild type. It is speculated that the *SsCI33130* gene may be involved in the development of sexual mating and pathogenicity in *S. scitamineum* by regulating the synthesis of the small-molecule signaling substances (cAMP or tryptophol) required during the sexual mating of *S. scitamineum*, thereby providing a molecular basis for the study of the pathogenic mechanisms of *S. scitamineum*.

## Introduction

Sugarcane is an important sugar crop and a potential renewable biomass energy crop. Sugarcane smut is a significant disease affecting sugarcane worldwide. The typical symptoms include black whip produced by the variation of the growing point of sugarcane, which causes serious damage to the normal growth of sugarcane and thus serious economic losses. Sugarcane smut was first found in KwaZulu-Natal, South Africa, in 1877 (McMartin, 1945). In China, it was first detected in Guangzhou in 1932 (Antoine, 1961). Since the 1990s, the occurrence of sugarcane smut has been increasing annually in China’s sugarcane producing areas, and in the past 20 years, it has become the most serious sugarcane disease in China (Shen et al., 2016a; Dou et al., 2017). At present, the main sugarcane cultivars in China are commonly infected with sugarcane smut, with a field incidence of 15–25%, and up to 50% of fields experience severe disease or even the loss of harvest (Shen et al., 2016b; Deng et al., 2020). Sugarcane smut is caused by *Sporisorium scitamineum*, which can be divided into a haploid spore stage and dikaryotic mycelium stage (Que et al., 2014). The teliospores of *S. scitamineum* are black or reddish-brown round asexual spores with papillae. The single spore has spinules on the surface and is about 5–6 μm in diameter (Wu et al., 2007). The temperature range of germination of the teliospores is 15–35°C, the optimum temperature is 28°C, the optimum pH range is from acidic to neutral, and the spores can die within 10 min at 55°C (Wu et al., 2010). Under suitable conditions, the teliospores can germinate and grow basidiospores of different lengths. Each basidiospore is composed of four oval haploid spores, two of which are the “+” mating type and two of which are the “−” mating type. The two opposite mating types of haploid spores can form white or yellow villous dikaryotic mycelia by sexual mating, which can infect the host sugarcane (Alexander and Srinivasan, 1966; Albert and Schenck, 1996; Yan et al., 2016a; Zhang et al., 2019).

*Sporisorium scitamineum* is a dimorphic fungus. The transformation from haploid to diploid is not only the transformation from yeast to mycelium but is also an important genetic change (Que et al., 2014). The pathogenicity of *S. scitamineum* is closely related to its sexual mating. The gene *b*E of the locus *b* of *S. scitamineum* is related to the sexual mating and pathogenicity of this pathogen. After the gene was knocked out, the sexual mating ability of WT17 and WT18 was completely lost, and they were unable to infect sugarcane (Yan et al., 2016b). Sun et al. (2019) found that the gene *Ram1* encoding the farnesyltransferase (FTase) β-subunit in *S. scitamineum* through gene knockout and gene complement techniques was related to sexual mating, cell wall stability, and pathogenicity in *S. scitamineum*. Zhu et al. (2020) reported that the pheromone response factor *SsPRF1* was involved in the regulation of mycelial growth, sexual mating, and pathogenicity in *S. scitamineum*. Chang et al. (2018) found that the cyclic adenosine monophosphate (cAMP)/protein kinase A (PKA) signaling pathway can regulate the intracellular reactive oxygen species (ROS) level, thereby regulating the sexual mating ability of *S. scitamineum*. Deng Y. Z. et al. (2018) found that the *SsKPP2* gene encoding mitogen-activated protein kinase (MAPK) in *S. scitamineum* affects the sexual mating and filamentation of *S. scitamineum* by regulating tryptophol biosynthesis and the pheromone signal transduction pathway.

The ubiquitin proteasome system (UPS) is one post-translational modification (PTM) mechanism. It is the main metabolic and protein regulatory system in eukaryotes. The ubiquitin molecule is a small protein composed of 76 amino acids and is common in eukaryotic cells (Goldstein et al., 1975). Protein ubiquitination is a process involving binding of ubiquitin to protein under the action of ubiquitin-activating enzyme E_1_, ubiquitin-binding enzyme E_2_, and ubiquitin ligase E_3_ (Finley et al., 1984). Deubiquitination (DUB) is the reverse process of protein ubiquitination by the removal of ubiquitin molecules from their binding substrates under the action of DUB enzymes. Deubiquitinases are mainly divided into six families, i.e., ubiquitin-specific processing proteases (UBP/USP), ubiquitin carboxyl-terminal hydrolases (UCH), the Ataxin-3/Josephin domains, the ovarian tumor domain-containing proteases (OUT), Machado-Joseph disease related enzymes (MJD), Jad1/Pad/MPN domain containing metallo enzymes (JAMM), and monocyte chemotactic protein-induced protein (MCPIP) (Fraile et al., 2012). Some studies on fungal deubiquitinases have been reported. The deletion of the gene *Moubp8* encoding UBP-deubiquitinase in *Magnaporthe oryzae* led to a deficiency in the vegetative growth and melanin synthesis of the pathogen, resulting in a significant decrease in conidia production and pathogenicity. In addition, the colonization ability of the mutant was significantly decreased by an osmotic colonization test (Yang et al., 2020). In *Saccharomyces cerevisiae*, the deletion of the gene *UBP14* encoding deubiquitinase results in a reduction of meiosis efficiency in *S. cerevisiae*, and the mutation of the *DOA4* gene encoding deubiquitinase affects the sporulation ability of the fungus (Swaminathan et al., 1999; Enyenihi and Saunders, 2003). In *Cryptococcus neoformans*, the deletion of the ubiquitinase *UBP5* gene results in obvious phenotypic defects and reduced pathogenicity (Wei et al., 2012). Cai et al. (2016) obtained a knockout mutant of the gene *Rcm1* (JAMM family) encoding a zinc finger protein in *S. scitamineum* through gene knockout technology, and functional verification indicated that this gene participated in the sexual mating of *S. scitamineum* by regulating the intracellular cAMP concentration. At present, studies on fungal deubiquitinases mainly focus on the UBP/USP family. OTU1-deubiquitinase belongs to the OTU family. There are few reports about the gene encoding OTU1-deubiquitinase and its function in fungi (Wei et al., 2012; Qin et al., 2021), but there are no reports about OTU1-deubiquitinase in *S. scitamineum*. It is thus necessary to study OTU1-deubiquitinase in *S. scitamineum*.

Based on the transcriptome sequencing data of the different pathogenic strains of *S. scitamineum* in our laboratory (Wu et al., 2020), we selected a gene *SsCI33130* (GenBank accession no. MZ408540) that encodes OTU1-deubiquitinase from differentially expressed genes with significantly upregulated expression levels in the strong pathogenic strain. In this study, polyethylene-glycol (PEG)-mediated protoplast transformation technology was used to obtain the gene *SsCI33130* knockout and complementation mutants. Through the phenotypic comparison of strain growth, morphology, abiotic stress, sexual mating, pathogenicity determination, and gene expression, the function of the gene *SsCI33130* in the sexual mating and pathogenicity of *S. scitamineum* was analyzed, so as to elucidate the molecular basis for the pathogenic mechanism of *S. scitamineum*.

## Results

### Identification and Characterization of *SsCI33130*

**FIGURE 1:**
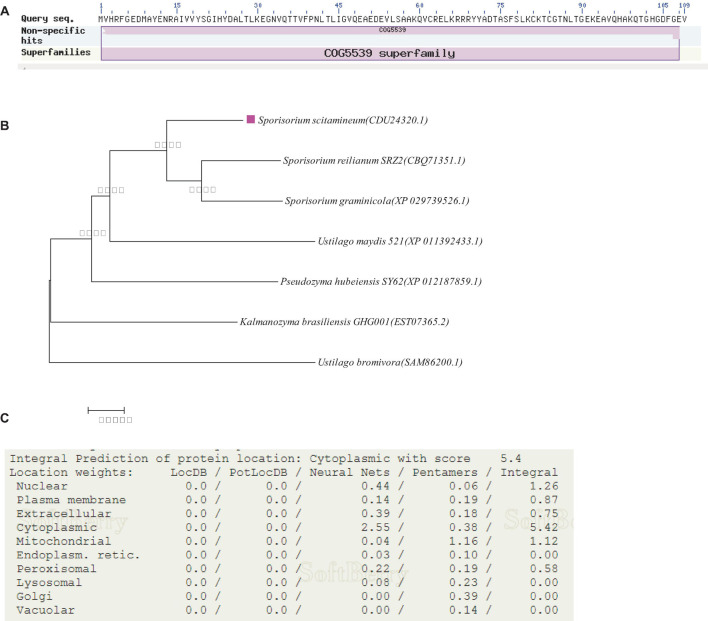
Identification and characterization of the *SSCI33130* gene. **(A)** Prediction of the protein encoded by the *SSCI33130* gene. The COG5539 superfamily indicated that the predicted protein encoded by the *SsCI33130* gene was OTU1-deubiquitinase (cysteine protease family). **(B)** Phylogenetic tree of the protein encoded by the *SsCI33130* gene. The rose-red square indicated that the *SsCI33130* gene of *S. scitamineum* was from this study. The protein accession number is in brackets. The credibility data obtained for 1000 iterations were calculated using bootstrapping, and the degree of credibility is marked by Arabic numerals beside the nodes on the tree. **(C)** Prediction of the subcellular localization of the protein encoded by the *SsCI33130* gene.

Through NCBI annotation, the predicted protein encoded by the gene *SsCI33130* is 100% similar to *S. scitamineum* OTU1-deubiquitylation enzyme and consists of 109 amino acid residues (NCBI: protein accession no. CDU24320.1). The isoelectric point (PI) of the protein was 6.06, the molecular weight (MW) was 12.16 kDa, the full length of the gene was 330 bp (Figure 1A). Phylogenetic analysis of the protein encoded by the *SsCI33130* gene showed that the protein encoded by the gene was highly conserved in smut fungi (Figure 1B). The subcellular localization prediction indicated that the protein encoded by the *SsCI33130* gene was localized in the cytoplasm of *S. scitamineum* (Figure 1C).

### Construction of *SsCI33130* Deletion and Complementary Mutants

**FIGURE 2:**
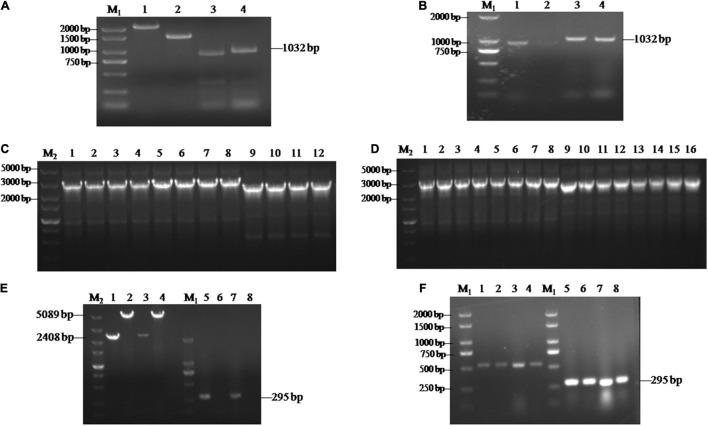
Construction and validation of the *SsCI33130* gene knockout and complementary mutants. Lane M_1_ is the 2000 marker, and lane M_2_ is the 5000 marker. **(A)** PCR amplification based on the wild type *Ss16*^+^. Lanes 1 and 2 represent the two overlapping *HPT* fragments with primer pairs Hpt-LB-F/226 and Hpt-RB-225/R, of which the band sizes were about 2 and 1.5 Kb, respectively. Lanes 3 and 4 represent the right and left borders of the *SsCI33130* gene amplified with primer pairs *SsCI33130*-RB-F/R and *SsCI33130*-LB-F/R. **(B)** PCR amplification based on the wild type *Ss16*^–^. Lanes 1 and 2 represent the right border of the *SsCI33130* gene amplified with primer pair *SsCI33130*-RB-F/R. Lanes 3 and 4 represent the left borders of the *SsCI33130* gene amplified with primer pair *SsCI33130*-LB-F/R. **(C)** Fusion-PCR based *Ss16*^+^. Lanes 1–8 represent the fusion fragments of the left borders of the wild type *Ss16*^+^ with the left *HPT* fragments, and lanes 9–12 represent the fusion fragments of the right borders of the wild type *Ss16*^+^ with the right *HPT* fragments. **(D)** Fusion-PCR based *Ss16*^–^. Lanes 1–8 represent the fusion fragments of the left borders of the wild type *Ss16*^–^ with the left *HPT* fragments, and lanes 9–16 represent the fusion fragments of the right borders of the wild type *Ss16*^–^ with the right *HPT* fragments. **(E)** The knockout mutants (Δ*Ss33130*^+^ and Δ*Ss33130*^–^) in the two mating-type backgrounds (*Ss16*^+^ and *Ss16*^–^) were confirmed by PCR. Lanes 1–4 represent the amplification result of the primer pair *SsCI33130*-OF/OR; lanes 5–8 represent the amplification result of the primer pair *SsCI33130*-IF/IR. Lanes 1 and 3 are the wild types *Ss16*^+^, *Ss16*^–^, respectively; lanes 2 and 4 are the knockout mutants Δ*Ss33130*^+^, Δ*Ss33130*^–^, respectively; lanes 5 and 7 are the wild types *Ss16*^+^, *Ss16*^–^, respectively; lanes 6 and 8 are the knockout mutants Δ*Ss33130*^+^, Δ*Ss33130*^–^, respectively. The amplified bands in lanes 1 and 3, lanes 2 and 4, and lanes 5 and 7 were 2408, 5089, and 295 bp, respectively. **(F)** Electrophoretic validation of the complementary mutants (*COM33130*^+^ and *COM33130*^–^) positive transformants. Lanes 1, 2, 5, and 6 were verified by complementary mutant electrophoresis in the Δ*Ss33130*^+^ background; lanes 3, 4, 7, and 8 were verified by the complementary mutant electrophoresis in the Δ*Ss33130*^–^ background; lanes 1–4 are the target gene fragment of zeocin amplified with primer pair Zeocin-JC-F/R; and lanes 5–8 are the *SsCI33130* target gene fragment amplified with primer pair *SsCI33130*-IF/IR.

To further investigate the functions of *SsCI33130*, gene deletion and complementary strains were constructed as described in the section “Materials and Methods.” Each flanking fragment was amplified by PCR with the primer pairs *SsCI33130*-LB-F/R and *SsCI33130*-RB-F/R. The band sizes were 917 and 1032 bp, respectively (Figures 2A,B). Then two fused fragments were amplified by PCR with the primer pairs *SsCI33130*-LB-F/Hpt-LB-226 and *SsCI33130*-RB-F/Hpt-RB-R. The band sizes were about 3 and 2.5 Kb, respectively (Figures 2C,D). They were used for *S. scitamineum* wild type protoplasts transformation. As expected, two positive deletion mutants Δ*Ss33130*^+^ and Δ*Ss33130*^–^ (Δ*Ss33130*^+^ is from the wild type *Ss16*^+^, Δ*Ss33130*^–^ is from the wild type *Ss16*^–^) were obtained. The *SsCI33130*-positive deletion mutants were confirmed by PCR using the primer pairs *SsCI33130*-OF/*SsCI33130*-OR and *SsCI33130*-IF/*SsCI33130*-IR. Use of the primer pair *SsCI33130*-IF/*SsCI33130*-IR resulted in a 295-bp band from the *SsCI33130* gene in the wild type (*Ss16*^+^ and *Ss16*^–^), but not in the deletion mutants, while the primer pair *SsCI33130*-OF/*SsCI33130*-OR produced a 5089-bp band in the deletion mutants and a 2408-bp band in the wild types, indicating that the gene *SsCI33130* was deleted in the wild type strains (*Ss16*^+^ and *Ss16*^–^) (Figure 2E). The *SsCI33130*-positive complementary mutants were confirmed with PCR using the primer pairs Zeocin-JC-F/Zeocin-JC-R (583 bp) and *SsCI33130*-IF/*SsCI33130*-IR (295 bp) (Figure 2F). Fortunately, two positive complementary mutants (*COM33130*^+^ and *COM33130*^–^) were obtained.

### Effects of the *SsCI33130* Gene on Abiotic Stress in *S. scitamineum*

**FIGURE 3:**
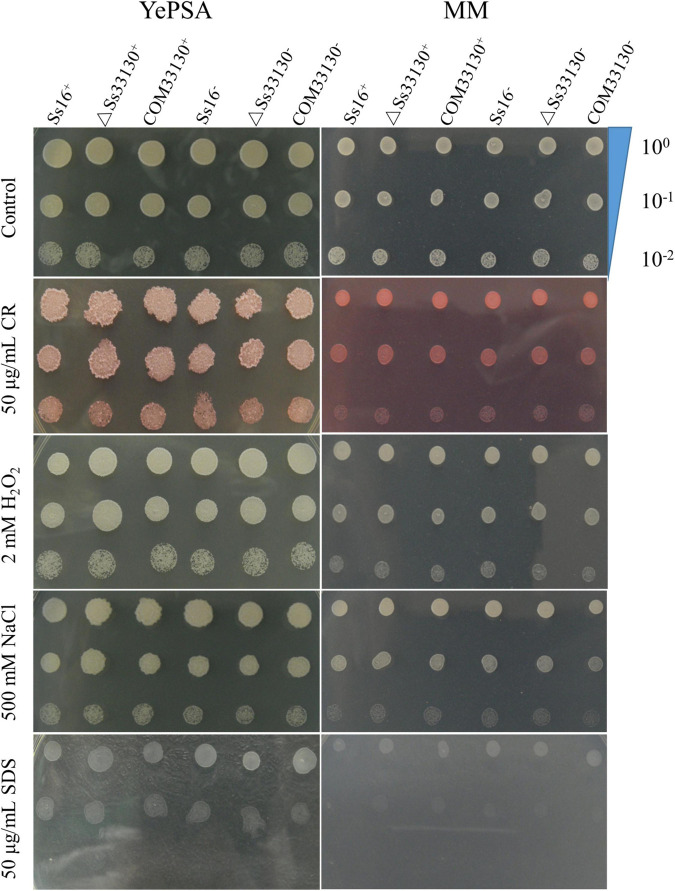
Effects of the *SsCI33130* gene on abiotic stress in *S. scitamineum*. Serially diluted cells of WT (*Ss16*^+^ and *Ss16*^–^), deletion mutants (Δ*Ss33130*^+^ and Δ*Ss33130*^–^), or complementary mutants (*COM33130*^+^ and *COM33130*^–^) were spotted onto YePSA or MM plates supplemented with H_2_O_2_ (2 mM), NaCl (500 mM), SDS (50 μg/mL), or Congo red (CR) (50 μg/mL). Samples were incubated at 28°C for 48 h before examination.

The response of the *SsCI33130* gene knockout mutants, complementary mutants, and wild type haploids to cell wall stress, osmotic stress, and reactive oxygen stress on solid plate medium was observed. The results showed that the phenotypes of the *SsCI33130* gene deletion mutants, complementary mutants, and their wild types were not significantly different on YePSA medium and MM medium (Figure 3). It is speculated that the *SsCI33130* gene is not involved in physiological functions such as oxidation, osmosis, and cell wall integrity.

### Effects of the *SsCI33130* Gene on the Growth and Morphology of *S. scitamineum*

**FIGURE 4:**
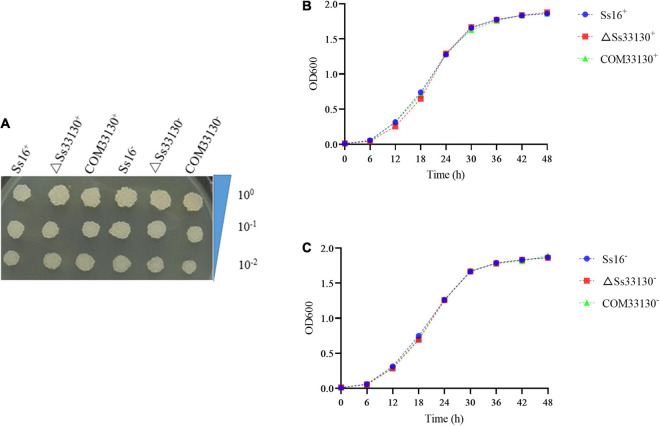
Effect of the *SsCI33130* gene on haploid spore growth in *S. scitamineum*. **(A)** Colony morphology of wild types, knockout mutants, and complementary mutants on YePSA medium (cultured at 28°C for 42 h). **(B)** Growth curve of the wild type *Ss16*^+^, knockout mutant Δ*Ss33130*^+^, and complementary mutant *COM33130*^+^ in YePS medium. **(C)** Growth curve of the wild type *Ss16*^–^, knockout mutant Δ*Ss33130*^–^, and complementary mutant *COM33130*^–^ in YePS medium.

There was almost no difference in the growth phenotype of the wild type, knockout mutant, and complementary mutant of *S. scitamineum* in YePSA solid medium (Figure 4A). In YePS liquid culture, the growth curves of the *SsCI33130* gene knockout mutant, complementary mutant, and wild type were basically the same (Figures 4B,C). The results showed that the *SsCI33130* gene did not affect the growth of *S. scitamineum*.

**FIGURE 5:**
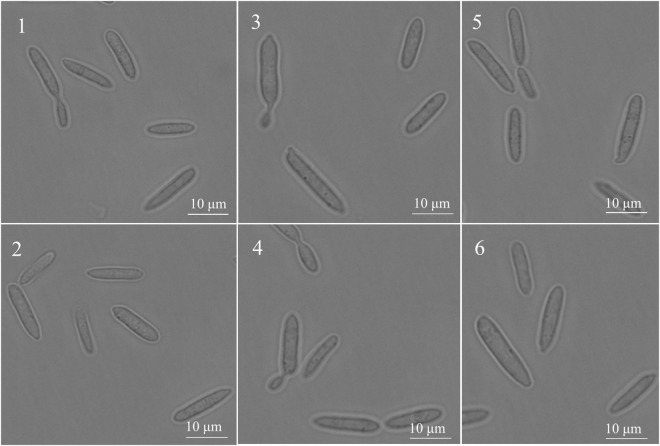
Effect of the *SSCI33130* gene on the haploid spore morphology of *S. scitamineum*. 1 and 2 are the spore morphology of wild types (*Ss16*^+^ or *Ss16*^–^) of *S. scitamineum*, respectively; 3 and 4 are the spore morphology of the *SSCI33130* knockout mutants (Δ*Ss33130*^+^ or Δ*Ss33130*^–^), respectively; 5 and 6 are the spore morphology of *SSCI33130* complementary mutants (*COM33130*^+^ or *COM33130*^–^), respectively.

Under the inverted fluorescence microscope (100 times oil microscope), the *SsCI33130* gene knockout mutant, complementary mutant, and wild type haploid were in the shape of one long rod or two long rods connected at the division stage (Figure 5), which indicated that the deletion of the gene did not affect the spore morphology of *S. scitamineum*.

### Effects of *SsCI33130* on Sexual Mating and the Expression of Genes Related to Signal Substance Synthesis

**FIGURE 6:**
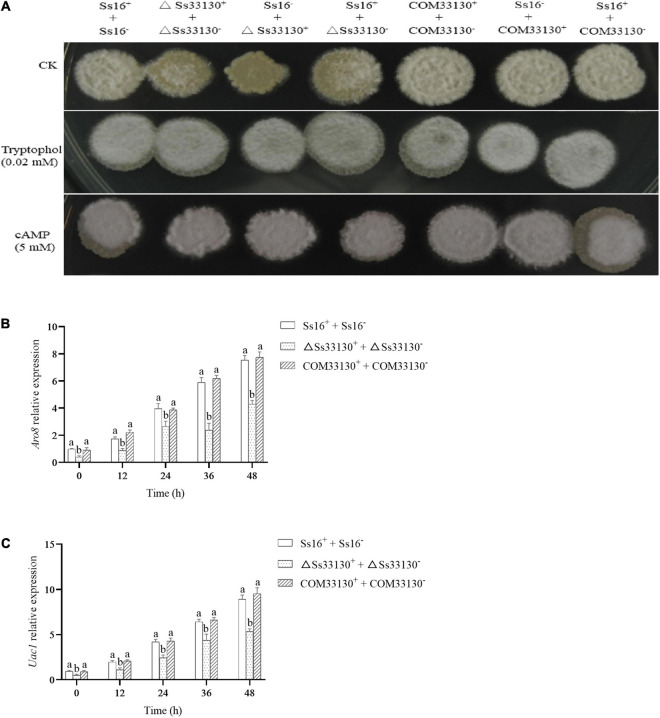
Determination of sexual mating ability and expression analysis of genes related to signal substance synthesis in *S. scitamineum*. **(A)** Comparison of sexual mating phenotypes. Sexual mating was assessed on YePSA plates supplemented with cAMP (5 mM) or tryptophol (0.02 mM). Photographs were taken 42 h after inoculation. The control was an untreated culture. **(B)** The expression level of the *Aro8* gene related to tryptophan synthesis. **(C)** The expression level of the *UAC1* gene related to cAMP biosynthesis. In the same group, different lowercase letters represent a difference at the 0.05 level.

The sexual mating between the wild types (*Ss16*^+^ + *Ss16*^–^) produced a large number of white villous hyphae, while the white villous hyphae produced between the knockout mutants (Δ*Ss33130*^+^ + Δ*Ss33130*^–^) and between the knockout mutant and wild type (*Ss16*^+^ + Δ*Ss33130*^–^, *Ss16*^–^ + Δ*Ss33130*^+^) were less than those produced between the wild types (*Ss16*^+^ + *Ss16*^–^), which indicated that the knockout mutants had weaker sexual mating ability. The sexual mating ability between the complementary mutants (*COM33130*^+^ + *COM33130*^–^) and between the complementary mutant and wild type (*Ss16*^+^ + *COM33130*^–^, *Ss16*^–^ + *COM33130*^+^) returned to the level of the wild type (*Ss16*^+^ + *Ss16*^–^) (Figure 6A). However, knockout of the *SsCI33130* gene did not affect the hyphal structure (Supplementary Figure 1). On the YePSA medium supplemented with 0.02 mM tryptophol or 5 mM cAMP, the sexual mating between the knockout mutants (Δ*Ss33130*^+^ + Δ*Ss33130*^–^) and between the wild types and the knockout mutants (*Ss16*^+^ + Δ*Ss33130*^–^, *Ss16*^–^ + Δ*Ss33130*^+^) was restored. It was thus speculated that this gene is involved in the sexual mating of *S. scitamineum* (Figure 6A). During 0–48 h of sexual mating, the expression level of the gene *ARO8* related to tryptophol biosynthesis in the knockout mutant combination (Δ*Ss33130*^+^ + Δ*Ss33130*^–^) was significantly lower than that in the wild type combination (*Ss16*^+^ + *Ss16*^–^) or complementary mutant combination (*COM33130*^+^ + *COM33130*^–^) (Figure 6B), while the expression level of the gene *ARO8* in the complementary mutant combination and in the wild type combination was almost the same (Figure 6B). Similarly, the expression level of the gene *UAC1* related to cAMP biosynthesis in the *SsCI33130* knockout mutant combination (Δ*Ss33130*^+^ + Δ*Ss33130*^–^) was significantly lower than that in the wild type combination (*Ss16*^+^ + *Ss16*^–^) and complementary mutant combination (*COM33130*^+^ + *COM33130*^–^), while the expression level of the gene *UAC1* was almost equal in the complementary mutant combination and wild type combination (Figure 6C). We hypothesized that the *SsCI33130* gene regulates the expression of genes related to the synthesis of the small molecule signaling substances (cAMP or tryptophol) required for the sexual mating of *S. scitamineum*, thereby affecting the synthesis of signaling substances.

### Effect of the *SSCI33130* Gene on the Activity of OTU1-Deubiquitinase in *S. scitamineum*

**FIGURE 7:**
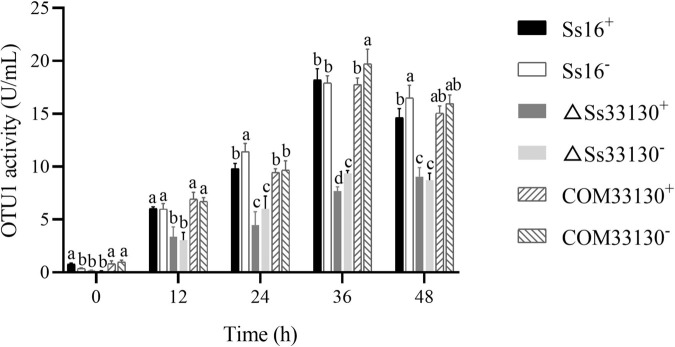
Comparison of OTU1-deubiquitinase activity of *S. scitamineum*. In the same group, different lowercase letters represent a difference at the 0.05 level.

Compared with the wild types (*Ss16*^+^ or *Ss16*^–^), the OTU1-deubiquitinase activity of the *SsCI33130* gene knockout mutants (Δ*Ss33130*^+^ or Δ*Ss33130*^–^) decreased significantly, and the OTU1-deubiquitinase activity of the *SsCI33130* gene complementary mutants (*COM33130*^+^ or *COM33130*^–^) was restored to the level of the wild type. In addition, the OTU1-deubiquitinase activities of the knockout mutants, complementary mutants, and wild types peaked at 36 h (Figure 7), indicating that the *SsCI33130* gene affected the OTU1-deubiquitinase activity of *S. scitamineum*.

### Expression Level of the *SsCI33130* Gene

**FIGURE 8:**
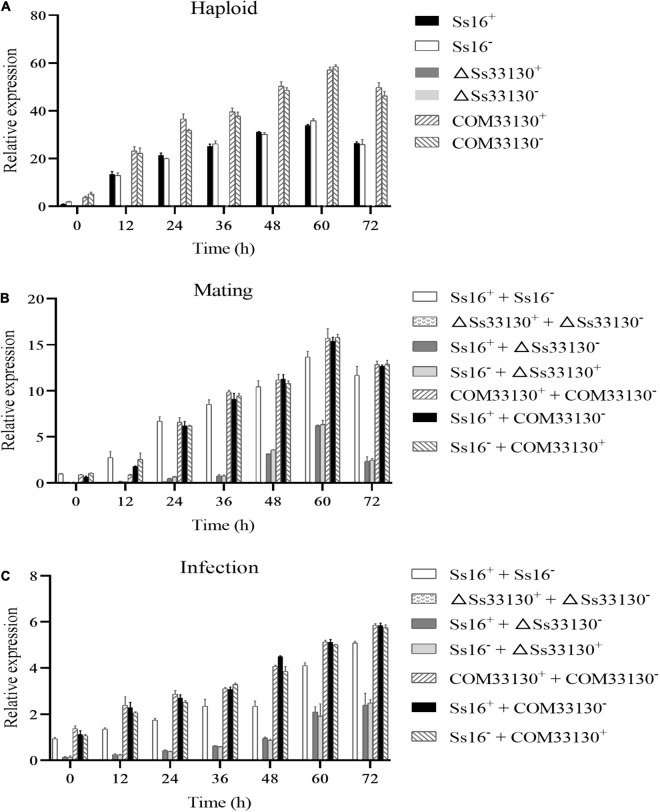
Expression analysis of the *SsCI33130* gene during various stages. **(A)** Gene expression levels under haploid sporidial growth. **(B)** Gene expression levels under sexual mating conditions. **(C)** Gene expression levels under sugarcane bud infection in the smut susceptible variety “ROC22”. *SsCI33130* gene expression at 0 h was normalized to one. Bars indicate the standard error. The analyses were repeated three times.

During the haploid spore culture process from 0 to 72 h, the expression level of the *SsCI33130* gene in the wild types or complementary mutants showed a trend of increasing first and then slightly decreasing, and the maximum expression level was observed at 60 h, but almost no expression was detected in the knockout mutants (Δ*Ss33130*^+^ or Δ*Ss33130*^–^) (Figure 8A). Similarly, in the process of sexual mating, the expression level of the *SsCI33130* gene showed a trend of increasing at first and then slightly decreasing, and the expression was the highest at 60 h, but almost no expression was detected between the knockout mutants (Δ*Ss33130*^+^ + Δ*Ss33130*^–^) (Figure 8B). In the process of sugarcane bud infection, the expression levels of this gene in the combinations containing wild types or complementary mutants showed gradually increasing trend, with the highest expression level detected at 72 h, but almost no expression was detected in the knockout mutant combination (Δ*Ss33130*^+^ + Δ*Ss33130*^–^) (Figure 8C). In the process of haploid spore culture, sexual mating, and sugarcane bud infection, the expression level of the *SsCI33130* gene in the complementary mutants returned to the wild type expression level and was even overexpressed (Figure 8).

### The *SsCI33130* Gene Is Involved in the Regulation of the Pathogenicity of *S. scitamineum*

**FIGURE 9:**
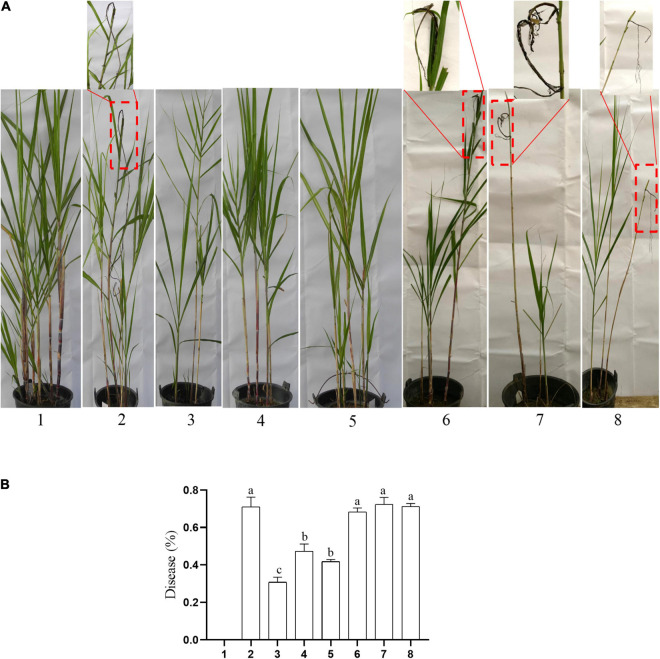
The *SsCI33130* gene involved in the pathogenicity of *S. scitamineum*. **(A)** Symptoms of smut whip. The symptoms in the box are magnified approximately 10 times. **(B)** Incidence: 1–8 are sterile water inoculation, *Ss16*^+^ + *Ss16*, Δ*Ss33130*^+^ + Δ*Ss33130*^–^, *Ss16*^+^ + Δ*Ss33130*^–^, *Ss16*^–^ + Δ*Ss33130*^+^, *COM33130*^+^ + *COM33130*^–^, *Ss16*^+^ + *COM33130*^–^, and *Ss16*^–^ + *COM33130*^+^, respectively. Different lowercase letters represent a difference at the 0.05 level.

The typical symptom of sugarcane smut is the formation of black whip from the growing point of the sugarcane plant. In this study, the symptoms of sugarcane smut were observed (Figure 9A), and the incidence rate of sugarcane smut was investigated (Figure 9B). The incidence rate of sugarcane smut between the knockout mutants (Δ*Ss33130*^+^ + Δ*Ss33130*^–^) and between the wild types and knockout mutants (*Ss16*^+^ + Δ*Ss33130*^–^, *Ss16*^–^ + Δ*Ss33130*^+^) was 30.77, 47.3, and 41.8%, respectively, which was significantly lower than the incidence rate (71.13%) between wild types (*Ss16*^+^ + *Ss16*^–^). The incidence rate of sugarcane smut between the complementary mutants (*COM33130*^+^ + *COM33130*^–^) and between the complementary mutant and wild type (*Ss16*^+^ + *COM33130*^–^, *Ss16*^–^ + *COM33130*^+^) was 68.33, 72.33, and 71.33%, respectively, which was not significantly different from the incidence rate (71.13%) between the wild types (*Ss16*^+^ + *Ss16*^–^). This suggested that the pathogenicity of the complementary mutants basically recovered to the level of the wild type.

## Discussion

In this study, we identified a gene, *SsCI33130*, encoding OTU1-deubiquitinase in the protein process of the endoplasmic reticulum pathway based on the transcriptome sequencing data of different pathogenic strains of *S. scitamineum* (Wu et al., 2020).

Phylogenetic tree analysis showed that the protein encoded by the *SsCI33130* gene was highly conserved in smut fungi, and subcellular localization prediction analysis showed that it was located in the cytoplasm. In this study, the activity of OTU1-deubiquitinase in *S. scitamineum* under haploid sporidia culture conditions was determined. It was found that the activity of OTU1-deubiquitinase in the knockout mutants was always significantly lower than that in the wild types, and the activity of OTU1-deubiquitinase in the complementary mutants returned to the wild type level, which verified the accuracy of the gene encoding OTU1-deubiquitinase. In this study, the *SsCI33130* gene knockout mutant exhibited no expression during haploid spore culture, sexual mating, or sugarcane bud infection, but the gene expression level of the complementary mutant was restored to the wild type level in the above process, which indicated that the knockout mutant and complementary mutant obtained in this study were effective, thus providing a foundation for further analysis.

The important role of protein DUB in eukaryotes is mainly reflected in the following aspects: correction, when the ubiquitin binding protein is not suitable, deubiquitinase removes ubiquitin from the target protein (Lam et al., 1997); maintenance of the normal rate of protein hydrolysis and the stability of the intracellular ubiquitin pool (Komander et al., 2009; Kimura and Tanaka, 2010); and the treatment of inactive ubiquitin precursors (Amerik and Hochstrasser, 2004). Protein DUB has important physiological functions and is involved in a variety of physiological activities in fungi. Protein DUB plays an important role in the vegetative growth, melanin synthesis, sporulation, colonization, and pathogenicity of fungi (Enyenihi and Saunders, 2003; Wei et al., 2012; Qin et al., 2021). The deletion of genes encoding deubiquitinase will lead to defects in fungal vegetative growth and melanin synthesis, as well as decreases in conidia production, colonization ability, and pathogenicity (Yang et al., 2020). In this study, the incidence rate of sugarcane smut in the combinations with *SsCI33130* knockout mutants (Δ*Ss33130*^+^ + Δ*Ss33130*^–^, *Ss16*^+^ + Δ*Ss33130*^–^, and *Ss16*^–^ + Δ*Ss33130*^+^) was significantly lower than that in the combinations without knockout mutants (*Ss16*^+^ + *Ss16*^–^, *COM33130*^+^ + *COM33130*^–^, *Ss16*^+^ + *COM33130*^–^, and *Ss16*^–^ + *COM33130*^+^). This indicates that the deletion of the gene *SsCI33130* will cause a significant decrease in the pathogenicity of *S. scitamineum*, confirming that deubiquitinase is involved in the pathogenicity of the fungus, which is consistent with previous findings (Enyenihi and Saunders, 2003; Wei et al., 2012; Yang et al., 2020). In addition, we found that the deletion of this gene did not affect the growth, morphology, abiotic stress, or other phenotypes of the haploid spores of *S. scitamineum*, which contrasts with the results reported by Wei et al. (2012) and Yang et al. (2020). The main reason may be that the functions of deubiquitinases in different families may differ. The results reported by Wei et al. (2012) and Yang et al. (2020) were mainly based on the deubiquitinases in the UBP/USP family, while the present study focused on the OTU1-deubiquitinases of the OTU family. Sebastian and Stefan (2006) found that the phenotype of the deletion mutant of the *otu1* gene encoding OTU1-deubiquitinase in yeast was not significantly different from that of the wild type, which was consistent with the fact that the deletion of the *SsCI33130* gene in this study did not cause phenotypic changes in the growth, morphology, and abiotic stress of *S. scitamineum*.

The pathogenicity of *S. scitamineum* is closely related to sexual mating. Haploid spores cannot produce hyphae and have no pathogenicity. Only the opposite mating-type haploid spores can form pathogenic dikaryotic mycelia through sexual mating. Therefore, sexual mating is the primary condition for the formation of dikaryotic mycelia and the development of pathogenicity in *S. scitamineum* (Que et al., 2014; Taniguti et al., 2015). In this study, the sexual mating ability between the *SsCI33130* gene deletion mutants (Δ*Ss33130*^+^ + Δ*Ss33130*^–^) and between the deletion mutants and wild type (*Ss16*^+^ + Δ*Ss33130*^–^, *Ss16*^–^ + Δ*Ss33130*^+^) was weaker than that between the wild types (*Ss16*^+^ + *Ss16*^–^), while the sexual mating ability between the complementary mutants (*COM33130*^+^ + *COM33130*^–^) and between the complementary mutant and wild type (*Ss16*^+^ + *COM33130*^–^, *Ss16*^–^ + *COM33130*^+^) returned to the level of the wild type. The pathogenicity and gene expression exhibited a similar phenomenon, which indicated that the deletion of *SsCI33130* affected the sexual compatibility and pathogenicity of *S. scitamineum*. Quorum-sensing substances play an important role in the pathogenicity of bacteria or fungi (Chen et al., 2004; Chen and Fink, 2006; Wongsuk et al., 2016). In this study, the exogenous addition of tryptophol or cAMP, a signaling substance required for sexual mating in *S. scitamineum* reported by previous researchers (Wang et al., 2019), could restore the sexual mating ability between the *SsCI33130* gene deletion mutants (Δ*Ss33130*^+^ + Δ*Ss33130*^–^) and between the deletion mutant and wild type (*Ss16*^+^ + Δ*Ss33130*^–^, *Ss16*^–^ + Δ*Ss33130*^+^) to the level of the wild type, indicating that the deletion of the gene affected the synthesis of signaling substances during sexual mating, such as tryptophol or cAMP, thus affecting the sexual mating ability of *S. scitamineum*.

In addition, we found that in the process of sexual mating, the expression levels of the genes *ARO8* or UAC1 related to tryptophol or cAMP synthesis in the *SsCI33130* knockout mutant combination (Δ*Ss33130*^+^ + Δ*Ss33130*^–^) were significantly lower than those in the wild type combination (*Ss16*^+^ + *Ss16*^–^), while the expression levels in the complementary mutant combination (*COM33130*^+^ + *COM33130*^–^) were restored to the wild type level, which showed that the deletion of the *SsCI33130* gene affected the expression of genes related to the synthesis of signaling substances, such as tryptophol or cAMP, and thus affected the synthesis of signaling substances. The *SsCI33130* gene encoded OTU1-deubiquitinase, which indicated that deubiquitinase was involved in the regulation of the sexual mating of *S. scitamineum*. Cai et al. (2016) found that *Rcm1*, a gene encoding a zinc-finger protein (JAMM family deubiquitinase), was involved in the sexual mating of *S. scitamineum* by regulating intracellular cAMP synthesis, indicating that deubiquitinase was involved in the sexual mating of *S. scitamineum*, which is consistent with the findings of this study. In addition, previous studies (Chang et al., 2018; Deng Y. Z. et al., 2018) reported that cAMP/PKA signaling pathway and tryptophol biosynthesis regulate the sexual mating and filamentation of *S. scitamineum*. Our study found that *SsCI33130* gene positively regulates the expression of genes related to cAMP or tryptophol biosynthesis. Therefore, the previous findings (Chang et al., 2018; Deng Y. Z. et al., 2018) are consistent with those of this study.

In conclusion, in this study, the gene *SsCI33130* encoding OTU1-deubiquitinase was identified based on the transcriptome data of differentially pathogenic *S. scitamineum* strains. The gene knockout and complementary mutants were successfully obtained by protoplast transformation technology. Pathogenicity related phenotypic comparisons among the gene knockout mutant, complementation mutant, and their wild type in terms of spore growth, morphology, abiotic stress, sexual mating ability, pathogenicity, and gene expression were conducted. The results showed that the gene had no effect on abiotic stress, cell wall integrity, spore growth, and morphology, but was related to the sexual mating and pathogenicity of *S. scitamineum*. The *SsCI33130* gene may affect the expression of genes related to the synthesis of small molecule signaling substances (cAMP or tryptophol) in the sexual mating of *S. scitamineum*, thus regulating the synthesis of signaling substances of sexual mating and participating in the development of the sexual mating and pathogenicity of *S. scitamineum*. This study provided a molecular basis for the sexual mating and pathogenicity of *S. scitamineum*.

## Materials and Methods

### Characterization of the *SsCI33130* Gene Sequence

Based on transcriptome sequencing data, a gene *SsCI33130* encoding OTU1-deubiquitinase was identified as a significantly (*P* ≤ 0.05) differentially expressed gene in haploids of the same mating type in isolates *Ss16* (strong pathogenic strain with 75% incidence rate of sugarcane smut in the susceptible variety “ROC22”) and *Ss47* (weak pathogenic strain with 25% incidence rate of sugarcane smut in the susceptible variety “ROC22”) of *S. scitamineum* with different pathogenicities (Wu et al., 2020). Blast comparison based on the amino acid sequences (DNA sequences into amino acid sequences)^1^ was performed on the NCBI database^2^ to obtain the conserved domain of the protein encoded by the *SsCI33130* gene. The *SsCI33130* encoding the protein sequence was analyzed using the Compute pI/M_w_ tool^3^ to determine the theoretical pI and MW. Phylogenetic analysis of the protein encoded by the *SsCI33130* gene was conducted in MEGA 7, and the phylogenetic tree was constructed using the neighbor-joining method (Saitou and Nei, 1987; Kumar et al., 2016). The ProComp 9.0 online tool^4^ was used to predict the subcellular localization of the *SsCI33130*-encoding protein.

### Fungal Isolates and Culture Conditions Used in This Study

The wild type haploid isolates *Ss16*^+^ and *Ss16*^–^ of *S. scitamineum* were isolated and identified in our laboratory and stored at −80°C (Deng Q. Q. et al., 2018). The culture media used in this study included YePSA medium (yeast extract 1%, peptone 2%, sucrose 2%, and agar 2%), YePS liquid medium (yeast extraction 1%, peptone 2%, sucrose 2%, pH 7.0), YePS soft medium (yeast extract 1%, peptone 2%, sugar 2%, agar 0.65%), YePSS medium (yeast extract 1%, peptone 2%, sugar 2%, D-sorbitol 18.17%, agar 2%), and MM medium (K_2_HPO_4_ 0.205%, KH_2_PO_4_ 0.145%, NH_4_NO_3_ 0.05%, (NH4)_2_SO_4_ 0.03%, FeSO_4_ 0.001%, CaCl_2_ 0.001%, Glucose 0.2%, 500 μL Z-Buffer, pH 6.7–7.0). For the mating/filamentation assay, equal volumes of wild type, deletion mutant, or complementary mutant haploid sporidia of opposite mating-types were mixed and plated on YePSA solid medium in the absence or presence of 5 mM cAMP and 0.02 mM tryptophol and then kept in the dark in a 28°C incubator for 42 h before photographing (Sun et al., 2019). For stress tolerance assessment, the sporidial culture at OD_600_ = 1.0 and its 10-fold serial dilutions were inoculated on YePSA medium or MM medium in the absence or presence of stress inducers, including 50 μg/mL Congo red (CR), 50 μg/mL SDS, 2 mM H_2_O_2_, and 500 mM NaCl, and then incubated in the dark at 28°C for 48 h before assessment and photographing. For the growth assay, the sporidia of wild type *S. scitamineum* (*Ss16*^+^ and *Ss16*^–^), deletion mutant (Δ*Ss72380*^+^ and Δ*Ss72380*^–^), and complementary mutant (*COM72380*^+^ and *COM72380*^–^) were cultured in 50 mL of YePS liquid medium at 28°C with shaking at 200 rpm for 24 h. Aliquots of cultured sporidia were then diluted with fresh YePS liquid medium, the cell density was adjusted to 10^5^ cells per mL, and samples were then cultured for another 48 h under the same conditions. The OD_600_ was measured with a spectrophotometer (NanoDrop 2000C) every 6 h to monitor the yeast-like (budding) growth of the wild type, deletion mutant, or complementary mutant strains.

### Nucleic Acid Manipulation

Fungal genomic DNA was extracted using a modified CTAB method (Shen et al., 2006). The PCR amplification was performed using Phanta High-Fidelity DNA Polymerase (Vazyme, P505). Purification of DNA fragments was conducted using a FastPure Gel DNA Extraction Mini Kit (Vazyme, DC301). Total RNA was extracted with TRIzol (Vazyme, R401), and HISCRIPT III RT SuperMix (Vazyme, R323) was used for cDNA synthesis. A NanoDrop ND-1000 (Thermo Fischer Scientific, Wilmington, DE, United States) was used for measuring the concentration and purity.

### Construction of *SsCI33130* Gene Knockout and Complementary Mutants

The construction of two fragments for the replacement of the *SsCI33130* gene by the Hpt (encodes a phosphotransferase conferring hygromycin resistance) gene was based on previous methods (Yang and Chung, 2012; Li et al., 2014; Chang et al., 2018). The flanking DNA of the *SsCI33130* gene was PCR-amplified using wild type *S. scitamineum* genomic DNA (*Ss16*^+^ and *Ss16*^–^). The Hpt gene in the plasmid pDAN (a circled vector) was the template. Gene knockout mutants were obtained by PEG-mediated protoplast transformation (Deng Y. Z. et al., 2018; Cai et al., 2020). All primers involved in the construction and validation of the knockout mutants are indicated in Table 1.

**TABLE 1:** Primers used in this study.

**Name**	**Primer sequences (5′-3′) Description**	
*SsCI33130*-LB-F	ACCCTTAGCCTTGGTATCGGGG	
*SsCI33130*-LB-R	GTCGTGACTGGGAAAACCCTGGAC ACCGAAGTGGT GTGAGAGGATCG	
*SsCI33130*-RB-F	GGTCATAGCTGTTTCCTGTGTGAG GAGAAGGAAG GAAGCTTGAATCG	Deletion construction
*SsCI33130*-RB-R	GGCGTCTTAAAGGGTTCGAGG AGTAG	
Hpt-LB-F	CAGGGTTTTCCCAGTCACGAC	
Hpt-LB-226	GGTCAAGACCAATGCGGAGC	
Hpt-RB-225	GCAAGACCTGCCTGAAACCG	
Hpt-RB-R	TCACACAGGAAACAGCTATGACC	
33130COM-F	ATCCAAGCTCAAGCTAAGCTTACCC TTAGCCTTGGTATCGGG CAGCAAGATCTAATCAAGCTTGGCG TCTTAAAGGGTTCGAGG	Complementation construction
33130COM-R	GCGCGCGTAATACGACTCAC GAAGTGCACGCAGTTGCCG	
COM-HPT-LB-F	CTCCGTGTTGATGCTGGGAC	
Zeocin-R Situ-F COM-HPT-RB-R	CGAGCATTCACTAGGCAACCA	
*SsCI33130*-IF	GTTTGGCGAGGACATGGCGTAT	PCR verification
*SsCI33130*-IR	ACCAGTCTGCTTGGCATGTT	
*SsCI33130*-OF	CCATAACGCCGAGCACCTTGAC	
*SsCI33130*-OR	GCTGGGCGTCTTAAAGGGTTCG	
Zeocin-JC-F	CGAGGTGGTTGCCCGTGTTT	
Zeocin-JC-R	CGGAAGTTCGTGGACACGAC	
*SsCI33130*-qF	ATTGTCGTCTACTCTGGCAT	qRT-PCR
*SsCI33130*-qR	GCATAGTACCTCCTCCTCTTTA	
*Aro8*-qF	CCTGGTGTTGCGTTCATTCC	
*Aro8*-qR	CAAGCTCGGGCATCGTCTTA	
*Uac1*-qF	CTGACGGAGATGTAGCCAAAG	
*Uac1*-qR	AACGAGACAAGGAGGGAGTA	
Actin-qF	ACAGGACGGCCTGGATAG	
Actin-qR	TCACCAACTGGGACGACA	

The complementation of the *SsCI33130* gene followed a previous strategy (Chang et al., 2018). The complemented gene not only carries the hygromycin homologous fragment to replace the hygromycin fragment in the knocked-out mutants but also carries the zeocin resistance marker gene to screen the complements. Complementary mutants were obtained by PEG-mediated protoplast methods (Chang et al., 2018; Cai et al., 2020). All primers involved in the construction and validation of the complementary mutants are listed in Table 1.

### OTU1-Deubiquitinase Activity Assay

We assessed the effect of the *SsCI33130* gene on OTU1-deubiquitinase activity every 12 h over a period of 48 h under haploid sporidia culture conditions (YePS liquid medium, 28°C, 200 rpm) based on the determination of the OTU1-deubiquitinase activity of the knockout mutants, complementary mutants, and wild types of the *SsCI33130* gene. The OTU1-deubiquitinase activity determination was conducted in accordance with the instructions of the fungal OTU1-deubiquitinase enzyme linked immunosorbent assay (ELISA) kit (Shanghai Enzyme-linked Biotechnology). This kit uses double antibody sandwich method to determine the level of fungal OTU1-deubiquitinase in samples.

### Analysis of Gene Expression

To evaluate the effect of *SsCI33130* on the expression of genes related to the synthesis of sexual mating signal substances in *S. scitamineum*, we used quantitative real-time (qRT)-PCR to detect the expression levels of the genes *Aro8* and *Uac1*, which are related to the synthesis of signaling substances (tryptophol and cAMP) (Chang et al., 2018; Wang et al., 2019). Three sexual mating combinations, i.e., a wild type combination (*Ss16*^+^ + *Ss16*^–^), knockout mutant combination (Δ*Ss33130*^+^ + Δ*Ss33130*^–^), and complementary mutant combination (*COM33130*^+^ + *COM33130*^–^), were designed. During the 0–48 h sexual mating process, samples were taken every 12 h, the total RNA of the samples was extracted, and gene expression was detected by qRT-PCR. For the qRT-PCR, we used a ChamQ Universal SYBR qPCR Master Mix (Vazyme, Q711), and the reaction was run on a real-time PCR system (CFX96, Bio-Rad). Relative expression values were calculated with the 2^–ΔΔCt^ method using *ACTIN* as an internal control (Livak and Schmittgen, 2001). Three biological repeats each containing three technical replicates for each sample were performed. The nucleotide sequences of primers *Aro8*-qF/qR, *Uac1*-qF/qR, and *ACTIN*-qF/qR are listed in Table 1.

We assessed the transcriptional profile of the *SsCI33130* gene every 12 h over a period of 72 h under haploid and mating conditions as well as during the infection process (after inoculation of the sugarcane buds of the smut susceptible variety “ROC22.” For detailed inoculation methods, refer to section “Assay of the Pathogenicity of the Knockout and Complement Mutants of the *SsCI33130* Gene” (section “Materials and Methods” below) using qRT-PCR. The method of qRT-PCR and the calculation of gene relative expression values were the same as those stated above. The nucleotide sequences of the primers *SsCI33130*-qF/qR and *ACTIN*-qF/qR are listed in Table 1.

### Assay of the Pathogenicity of the Knockout and Complement Mutants of the *SsCI33130* Gene

Sporidial colonies of the wild types (*Ss16*^+^ and *Ss16*^–^), knockout mutants (Δ*Ss33130*^+^ and Δ*Ss33130*^–^), or complementary mutants (*COM33130*^+^ and *COM33130*^–^) were inoculated into 50 mL of YePS liquid medium and cultured at 28°C with shaking at 200 rpm for 2 days. Sporidia were collected by centrifugation and washed twice with ddH_2_O, after which they were re-suspended in YePS liquid medium at a final concentration of 2 × 10^9^ spores/mL. Sporidia of opposite mating types were then mixed in equal volumes, after which 200 μL of this mixture was syringe-injected into growth point of seedlings with 4–5 leaves from the highly susceptible sugarcane variety “ROC22” (20 plants were inoculated in each treatment) (Shen et al., 2014; Chang et al., 2018). A wild type mixture (*Ss16*^+^ and *Ss16*^–^) served as a positive control, and sterile water was used as a negative control. Inoculated plants were kept in a greenhouse (30°C, relative humidity 70%) for 4 months. Investigation of the occurrence of sugarcane smut began after 1 month. At the end of each investigation, the diseased plants were labeled to avoid repeated investigations, black whip symptoms were covered with plastic bags to prevent the spread of teliospores, and the number of diseased plants (including anatomically confirmed plants with early symptoms of smut) and the morbidity were calculated.

### Microscopy

Images were taken using an Axio Observer Z1 microscope (Zeiss, Jena, Germany) equipped with a sCMOS camera (PCO Edge, Kelheim, Germany).

### Statistic Analysis

Data were expressed as the means ± standard error (SE). Differences among different treatments were analyzed using GraphPad Prism 8 software (GraphPad, United States).

## Data Availability Statement

The datasets presented in this study can be found in online repositories. The names of the repository/repositories and accession number(s) can be found below: https://www.ncbi.nlm.nih.gov/genbank/, BankIt2472583 SsCI33130MZ408540.

## Author Contributions

WS conceived and designed the experimental plan. HL, YC, and HB performed the experiments. HL YC, and WS analyzed the data and wrote the manuscript. WS, YC, HL, QD, and JC revised the manuscript. All authors read and approved the final version of the manuscript.

## Conflict of Interest

The authors declare that the research was conducted in the absence of any commercial or financial relationships that could be construed as a potential conflict of interest.

## Publisher’s Note

All claims expressed in this article are solely those of the authors and do not necessarily represent those of their affiliated organizations, or those of the publisher, the editors and the reviewers. Any product that may be evaluated in this article, or claim that may be made by its manufacturer, is not guaranteed or endorsed by the publisher.

## Footnotes

1
https://www.novopro.cn/tools/translate.html

2
https://blast.ncbi.nlm.nih.gov/Blast.cgi?PROGRAM=blastp&PAGE_TYPE=BlastSearch&LINK_LOC=blasthome

3
https://web.expasy.org/compute_pi

4
http://linux1.softberry.com/berry.phtml?topic=protcompan&group=programs&subgroup=proloc

## Supplementary Material

Supplementary Figure 1 Microscopic observation of mycelial structure of *S. scitamineum*. Photographs were taken 42 h after inoculation.

## References

[B1] Albert H. H. Schenck S. (1996). PCR amplification from a homolog of the *b*E mating-type gene as a sensitive assay for the presence of *Ustilago scitaminea* DNA. *Plant Dis.* 80 1189–1192. 10.1094/PD-80-1189

[B2] Alexander K. C. Srinivasan K. V. (1966). Sexuality in *Ustilago Scitaminea* syd. *Curr. Sci.* 35 603–604.

[B3] Amerik A. Y. Hochstrasser M. (2004). Mechanism and function of deubiquitinating enzymes. *Biochim. Biophys. Acta* 1695 189–207. 10.1016/j.bbamcr.2004.10.003 15571815

[B4] Antoine R. (1961). “Smut,” in *Sugarcane Disease of the World*, eds Martin J. P. Abbott E. V. Hughes C. G. (Amsterdam: Elsevier Publishing Company), 327–354.

[B5] Cai E. P. Mei D. Zhang X. M. Sun X. Li L. Y. Wu R. R. (2020). A gene knockout method based on protoplast transformation with two PCR fragments in *Sporisorium scitamineum*. *Mycosystema* 39 2314–2327. 10.13346/j.mycosystema.200273

[B6] Cai E. P. Yan M. X. Mei D. (2016). “Study on the role of zinc finger protein *RCM1* in sexual cooperation of *Sporisorium scitamineum*,” in *Annual Conference of Chinese Society of Mycology, Fuzhou, Fujian Province, China*, (Fujian Province: Chinese Society of Mycology).

[B7] Chang C. Q. Cai E. P. Deng Y. Z. Mei D. Qiu S. X. Chen B. S. (2018). cAMP/PKA signalling pathway regulates redox homeostasis essential for *Sporisorium scitamineum* mating/filamentation and virulence. *Environ. Microbiol.* 21 959–971. 10.1111/1462-2920.14496 30537399

[B8] Chen H. Fink G. R. (2006). Feedback control of morphogenesis in fungi by aromatic alcohols. *Genes Dev.* 20 1150–1161. 10.1101/gad.1411806 16618799 PMC1472474

[B9] Chen H. Fujita M. Feng Q. Clardy J. Fink G. R. (2004). Tyrosol is a quorumsensing molecule in *Candida albicans*. *Proc. Natl. Acad. Sci.* 101 5048–5052. 10.1073/pnas.0401416101 15051880 PMC387371

[B10] Deng Q. Q. Wu J. Chen J. W. Shen W. K. (2020). Physiological mechanisms of improved smut resistance in sugarcane through application of silicon. *Front. Plant Sci.* 11:568130. 10.3389/fpls.2020.568130 33224161 PMC7674639

[B11] Deng Q. Q. Xu G. H. Dou Z. M. Shen W. K. (2018). Identification of three *Sporisorium scitamineum* pathogenic races in mainland China. *Int. J. Agric. Biol.* 20 799–802. 10.17957/IJAB/15.0566 29653435

[B12] Deng Y. Z. Zhang B. Chang C. Q. Wang Y. X. Lu S. Sun S. Q. (2018). The MAP kinase *SsKpp2* is required for mating/filamentation in *Sporisorium scitamineum*. *Front. Microbiol.* 9:2555. 10.3389/fmicb.2018.02555 30416495 PMC6212578

[B13] Dou Z. M. Deng Q. Q. Shen W. K. (2017). Evaluation of BC_3_F_1_ lines from intergeneric cross between *Erianthus arundinaceus* and *Saccharum* for resistance to sugarcane smut caused by *Sporisorium scitamineum*. *Int. J. Agric. Biol.* 19 1520–1524. 10.17957/IJAB/15.0457 29653435

[B14] Enyenihi A. H. Saunders W. S. (2003). Large-scale functional genomic analysis of sporulation and meiosis in Saccharomyces cerevisiae. *Genetics* 163 47–54. 10.1023/A:1022320801661 12586695 PMC1462418

[B15] Finley D. Ciechanover A. Varshavsky A. (1984). Thermolability of ubiquitin-activating enzyme from the mammalian cell cycle mutant ts85. *Cell* 37 43–55. 10.1016/0092-8674(84)90299-X 6327059

[B16] Fraile J. M. Quesada V. Lopez O. C. (2012). Deubiquitinases in cancer:new functions and therapeutic options. *Oncogene* 31 2373–2388. 10.1038/onc.2011.443 21996736

[B17] Goldstein G. Scheid M. Hammerling U. (1975). Isolation of a polypeptide that has lymphocyte-differentiating properties and is probably represented universally in living cells. *Proc. Natl. Acad. Sci.* 72 11–15. 10.1073/pnas.72.1.11 1078892 PMC432229

[B18] Kimura Y. Tanaka K. (2010). Regulatory mechanisms involved in the control of ubiquitin homeostasis. *Biochem* 147 793–798. 10.1093/jb/mvq044 20418328

[B19] Komander D. Clague M. J. Urbe S. (2009). Breaking the chains: structure and function of the deubiquitinases. *Nat. Rev. Mol. Cell Biol.* 10 550–563. 10.1038/nrm2731 19626045

[B20] Kumar S. Stecher G. Tamura K. (2016). MEGA7: molecular evolutionary genetics analysis version 7.0 for bigger datasets. *Mol. Biol. Evol.* 33 1870–1874. 27004904 10.1093/molbev/msw054 PMC8210823

[B21] Lam Y. A. Xu W. DeMartino G. N. (1997). Editing of ubiquitin conjugates by an isopeptidase in the 26S proteasome. *Nature* 385 737–740. 10.1038/385737a0 9034192

[B22] Li M. H. Xie X. L. Lin X. F. Shi J. X. Ding Z. J. Ling J. F. (2014). Functional characterization of the gene *FoOCH1* encoding a putative α-1,6-mannosyltransferase in *Fusarium oxysporum f. sp. cubense*. *Fungal Genet. Biol.* 65 1–13. 10.1016/j.fgb.2014.01.005 24503549

[B23] Livak K. J. Schmittgen T. D. (2001). Analysis of relative gene expression data using realtime quantitative PCR and the 2^–ΔΔCt^ method. *Methods* 25 402–408. 10.1006/meth.2001.1262 11846609

[B24] McMartin A. (1945). Sugarcane smut:reappearance in Natal. *South Afri. Sugar J.* 29 55–57.

[B25] Qin L. Li X. W. Li D. Zhao J. R. Wang S. H. Yuan J. (2021). Protein kinase *Cla4* regulates morphology development, aflatoxin biosynthesis and pathogenicity of *Aspergillus flavus*. *J. Fungus* 40 174–188. 10.13346/j.mycosystema.200199

[B26] Que Y. X. Xu L. Wu Q. B. Liu Y. F. Ling H. Liu Y. H. (2014). Genome sequencing of *Sporisorium scitamineum* provides insights into the pathogenic mechanisms of sugarcane smut. *BMC Genomics* 15:996. 10.1186/1471-2164-15-996 25406499 PMC4246466

[B27] Saitou N. Nei M. (1987). The neighbor-joining method: a new method for reconstructing phylogenetic trees. *Mol. Biol. Evol.* 4 406–425. 10.1093/oxfordjournals.m-olbev.a040454 3447015

[B28] Sebastian R. Stefan J. (2006). Functional division of substrate processing cofactors of the ubiquitin-selective *Cdc48* chaperone. *Mol. Cell.* 21 261–269. 10.1016/j.molcel.2005.12.014 16427015

[B29] Shen W. K. Jiang Z. D. Yang Z. D. Liu R. Chen J. W. Deng H. H. (2014). New resistance identification method and resistance evaluation of sugarcane varieties to smut disease. *J. Huazhong Agric. Univ.* 33 51–56. 10.3969/j.issn.1000-2421.2014.02.009

[B30] Shen W. K. Xu G. H. Luo M. Z. Jiang Z. D. (2016a). Genetic diversity of *Sporisorium scitamineum* in Mainland China assessed by SCoT analysis. *Trop. Plant Pathol.* 41 288–296. 10.1007/s40858-016-0099-z

[B31] Shen W. K. Xu G. H. Sun L. H. Zhang L. H. Jiang Z. D. (2016b). Development of a Loop-Mediated Isothermal amplification assay for rapid and sensitive detection of *Sporisorium scitamineum* in sugarcane. *Ann. Appl. Biol.* 168 321–327. 10.1111/aab.12264

[B32] Shen W. K. Zhou G. H. Deng H. H. Zhou L. Y. (2006). Detection of sugarcane ratoon stunting disease pathogen with polymerase chain reaction(PCR) and nucleotide sequence analysis. *Chinese Agric. Sci. Bull.* 22 413–413. 10.3969/j.issn.1000-6850.2006.12.098

[B33] Sun S. Q. Deng Y. Z. Cai E. P. Yan M. X. Jiang Z. D. (2019). The Farnesyltransferase β-subunit *Ram1* regulates *Sporisorium scitamineum* mating, pathogenicity and cell wall integrity. *Front. Microbiol.* 10:976. 10.3389/fmicb.2019.00976 31134021 PMC6517510

[B34] Swaminathan S. Amerik A. Y. Hochstrasser M. (1999). The *Doa4* deubiquitinating enzyme is required for ubiquitin homeostasis in yeast. *Mol. Biol. Cell.* 10 2583–2594. 10.1091/mbc.10.8.2583 10436014 PMC25490

[B35] Taniguti L. M. Schaker P. D. C. Benevenuto J. (2015). Complete genome sequence of *Sporisorium scitamineum* and biotrophic interaction transcriptome with sugarcane. *PLoS One* 10:e0129318. 10.1371/journal.pone.0129318 26065709 PMC4466345

[B36] Wang Y. Deng Y. Z. Cui G. B. Huang C. W. Zhang B. Chang C. Q. (2019). The AGC Kinase *SsAgc1* regulates *Sporisorium scitamineum* mating/filamentation and pathogenicity. *mSphere* 4 e259–e219. 10.1128/mSphere.00259-19 31142621 PMC6541736

[B37] Wei F. Michael S. P. Dena L. T. (2012). Pleiotropic effects of deubiquitinating enzyme *Ubp5* on growth and pathogenesis of *Cryptococcus neoformans*. *PLoS One* 7:e38326. 10.1371/journal.pone.0038326 22719877 PMC3375289

[B38] Wongsuk T. Pumeesat P. Luplertlop N. (2016). Fungal quorum sensing molecules: role in fungal morphogenesis and pathogenicity. *J. Basic Microbiol.* 56 440–447. 10.1002/jobm.201500759 26972663

[B39] Wu H. W. Li R. He C. P. (2007). Preliminary investigation on sugarcane disease species in Hainan Island. *J. Trop. Crops* 28 112–116. 10.3969/j.issn.1000-2561.2007.04.023

[B40] Wu H. W. Zheng X. L. Li R. (2010). Basic biological characteristics of *Sporisorium scitamineum*. *J. Trop. Crops* 31 1388–1392.

[B41] Wu J. Li H. Z. Deng Q. Q. Chen J. W. Shen W. K. (2020). Transcriptomic analysis of *Sporisorium scitamineum* isolates with different pathogenicity. *J. Huazhong Agric. Univ.* 39 40–44. 10.13300/j.cnki.hnlkxb.2020.03.007

[B42] Yan M. X. Cai E. P. Zhou J. N. Chang C. Q. Xi P. G. Shen W. K. (2016a). A dual-color imaging system for sugarcane smut fungus *Sporisorium scitamineum*. *Plant Dis.* 100 2357–2362. 10.1094/PDIS-02-16-0257-SR 30686163

[B43] Yan M. X. Zhu G. N. Lin S. Y. Xian X. Y. Chang C. Q. Xi P. G. (2016b). The mating-type locus *b* of the sugarcane smut *Sporisorium scitamineum* is essential for mating, filamentous growth and pathogenicity. *Fungal Genet. Biol.* 86 1–8. 10.1016/j.fgb.2015.11.005 26563415

[B44] Yang J. Chen D. J. Matar K. (2020). The deubiquitinating enzyme *MoUbp8* is required for infection-related development, pathogenicity, and carbon catabolite repression in *Magnaporthe oryzae*. *Appl. Microbiol. Biotech.* 104 5081–5094. 10.1007/s00253-020-10572-5 32274561

[B45] Yang S. L. Chung K. R. (2012). The NADPH oxidase-mediated production of hydrogen peroxide (H_2_O_2_) and resistance to oxidative stress in the necrotrophic pathogen *Alternaria alternata* of citrus. *Mol. Plant Pathol.* 13 900–914. 10.1111/j.1364-3703.2012.00799.x 22435666 PMC6638813

[B46] Zhang B. Cui G. B. Chang C. Q. Wang Y. X. Zhang H. Y. Chen B. S. (2019). The autophagy gene *ATG8* affects morphogenesis and oxidative stress tolerance in *Sporisorium scitamineum*. *J. Integr. Agr.* 18 1024–1034.

[B47] Zhu G. Deng Y. Z. Cai E. P. Yan M. X. Cui G. B. Wang Z. Q. (2020). Identification and functional analysis of the pheromone response factor gene of *Sporisorium scitamineum*. *Front. Microbiol.* 10:2115. 10.3389/fmicb.2019.02115 31552011 PMC6747018

