# Supplementary material for: Ovarian Tumor Domain-Containing Proteases-Deubiquitylation Enzyme Gene SsCI33130 Involved in the Regulation of Mating/Filamentation and Pathogenicity in Sporisorium scitamineum
Source: Front Microbiol. 2021 Oct 5;12:746550. doi: 10.3389/fmicb.2021.746550 (PMC8523855; doi:10.3389/fmicb.2021.746550)

We blasted the sequence of *SsCI33130* encoding protein on NCBI (<https://blast.ncbi.nlm.nih.gov/Blast.cgi?PROGRAM=blastp&PAGE_TYPE=BlastSearch&LINK_LOC=blasthome>). The results showed that the gene encodes an OTU1-deubiquitylation enzyme in *Sporisorium scitamineum*. The website showed the prediction diagram of the conserved domain (**Figure 1A in the manuscript)**.


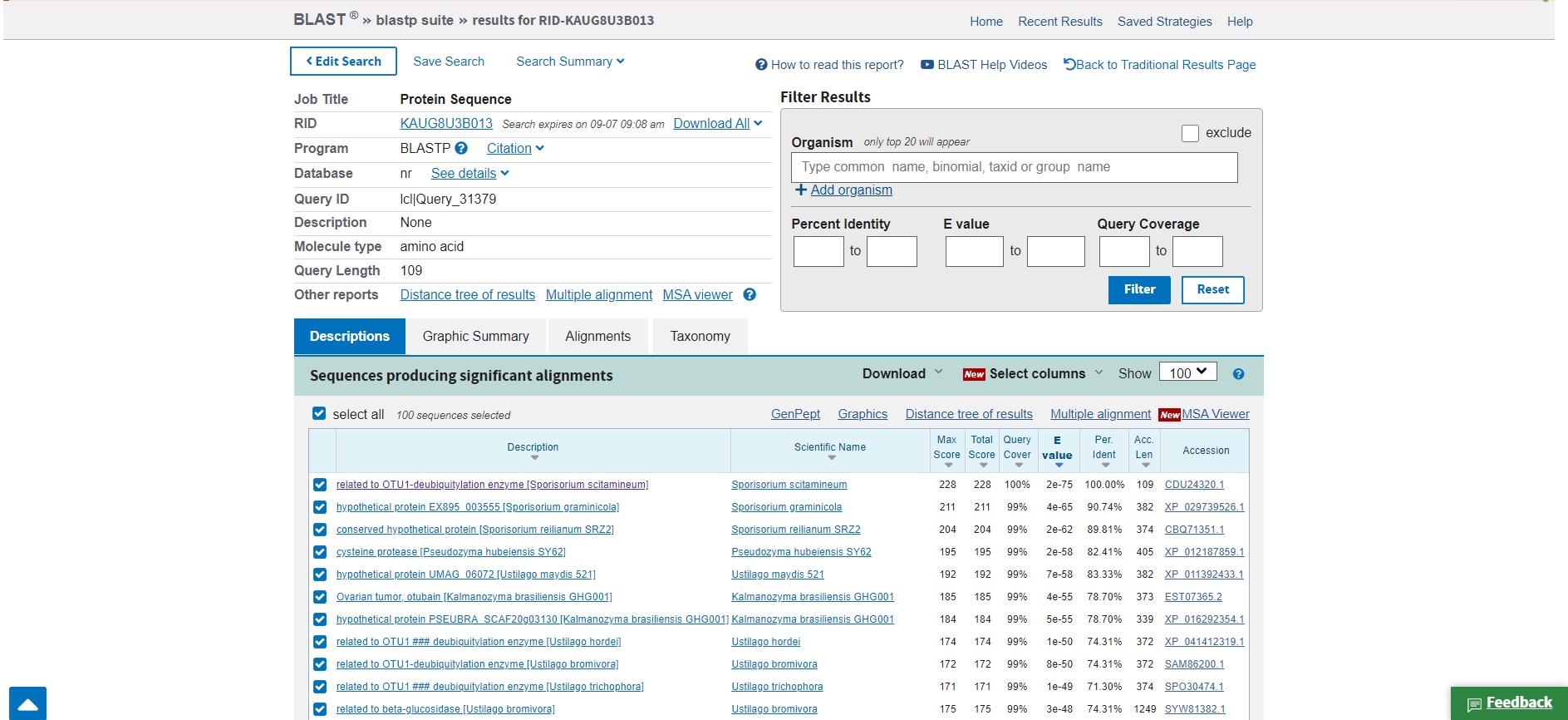

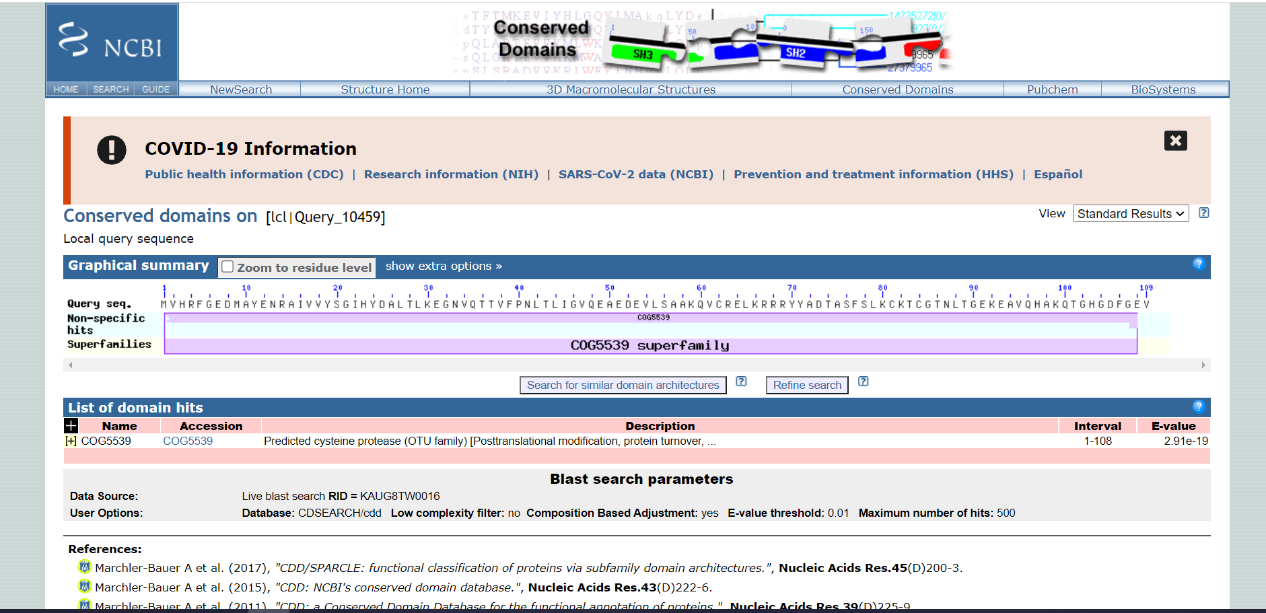


We also selected several protein sequences with the highest coverage rate to construct the phylogenetic tree using neighbor-joining method in MEGA 7. Phylogenetic tree showed that the gene was highly conserved (**Figure 1B in the manuscript**).

Sequence ID: [CDU24320.1](https://www.ncbi.nlm.nih.gov/protein/CDU24320.1?report=genbank&log$=protalign&blast_rank=1&RID=KAUG8U3B013)

[XP_029739526.1](https://www.ncbi.nlm.nih.gov/protein/XP_029739526.1?report=genbank&log$=protalign&blast_rank=2&RID=KAUG8U3B013)

[CBQ71351.1](https://www.ncbi.nlm.nih.gov/protein/CBQ71351.1?report=genbank&log$=protalign&blast_rank=3&RID=KAUG8U3B013)

[XP_012187859.1](https://www.ncbi.nlm.nih.gov/protein/XP_012187859.1?report=genbank&log$=protalign&blast_rank=4&RID=KAUG8U3B013)

[XP_011392433.1](https://www.ncbi.nlm.nih.gov/protein/XP_011392433.1?report=genbank&log$=protalign&blast_rank=5&RID=KAUG8U3B013)

[EST07365.2](https://www.ncbi.nlm.nih.gov/protein/EST07365.2?report=genbank&log$=protalign&blast_rank=6&RID=KAUG8U3B013)

[SAM86200.1](https://www.ncbi.nlm.nih.gov/protein/SAM86200.1?report=genbank&log$=protalign&blast_rank=9&RID=KAUG8U3B013)

Finally we predicted the subcellular localization of the *SsCI33130* encoding protein using the ProComp 9.0 online tool.

(<http://linux1.softberry.com/berry.phtml?topic=protcompan&group=programs&subgroup=proloc>)

The results showed that the predicted location of the protein is in the cytoplasm.


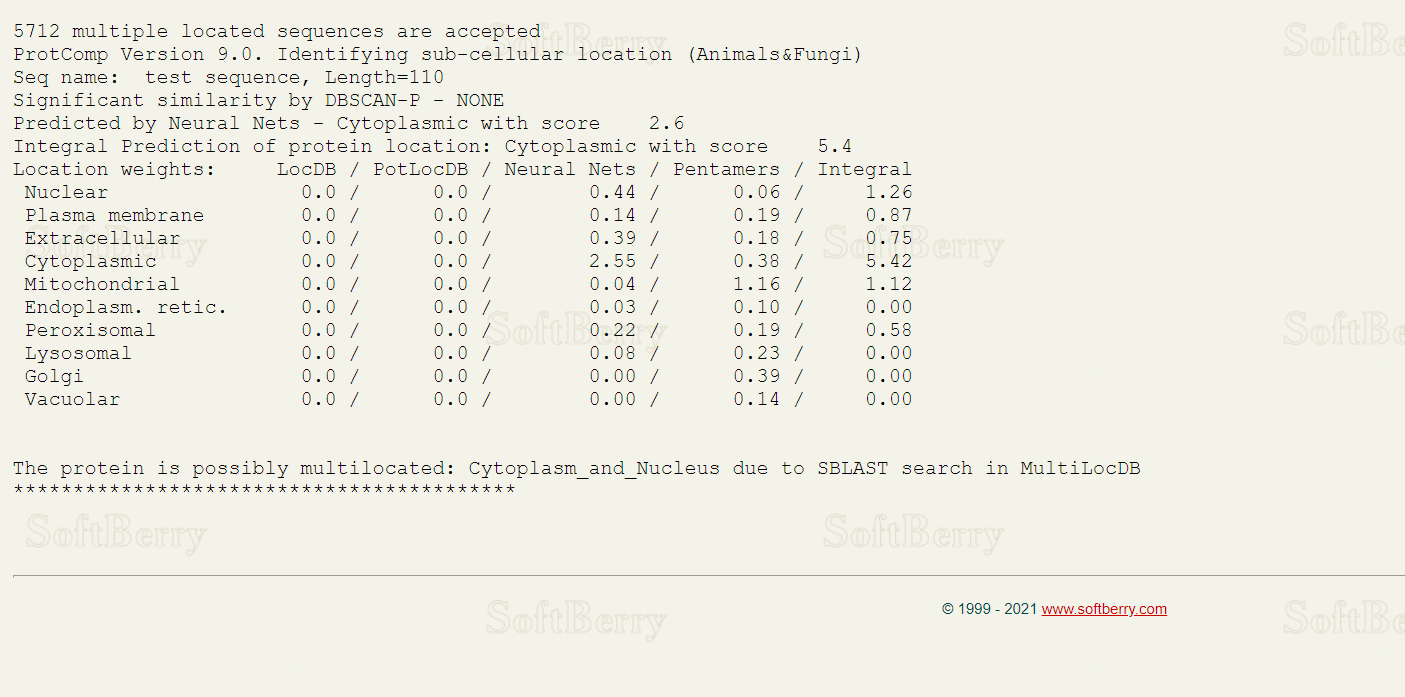

Supplement: Supplementary Figure 1 — Microscopic observation of mycelial structure of S. scitamineum. Photographs were taken 42 h after inoculation. [file Data_Sheet_1.zip › Figure 1 procedure.docx]
