# Supplementary figures and images for: Ovarian Tumor Domain-Containing Proteases-Deubiquitylation Enzyme Gene SsCI33130 Involved in the Regulation of Mating/Filamentation and Pathogenicity in Sporisorium scitamineum
Source: Front Microbiol. 2021 Oct 5;12:746550. doi: 10.3389/fmicb.2021.746550 (PMC8523855; doi:10.3389/fmicb.2021.746550)

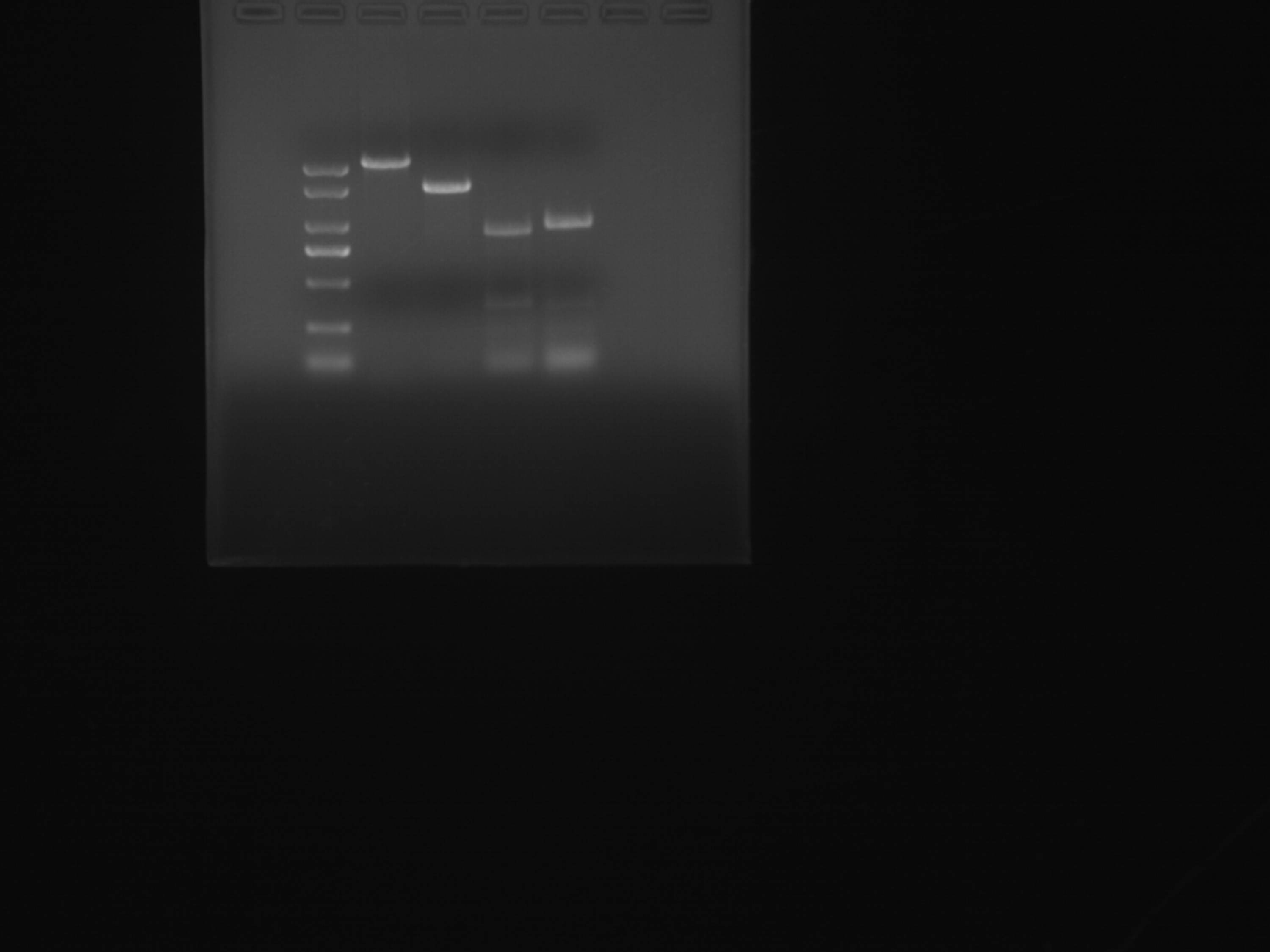

Supplement: Supplementary Figure 1 — Microscopic observation of mycelial structure of S. scitamineum. Photographs were taken 42 h after inoculation. [file Data_Sheet_1.zip › Figure 2 original images of gels/A.TIF]

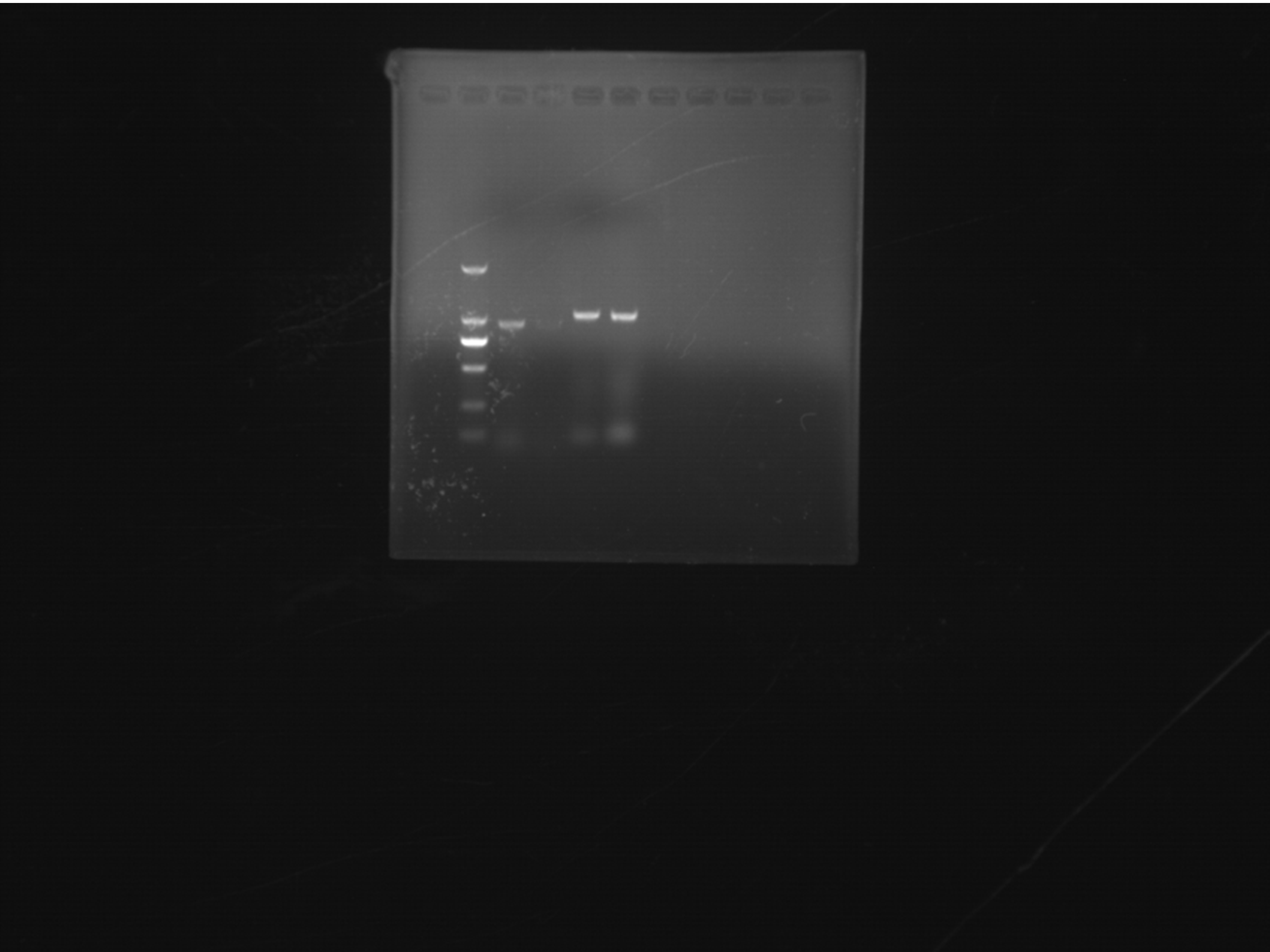

Supplement: Supplementary Figure 1 — Microscopic observation of mycelial structure of S. scitamineum. Photographs were taken 42 h after inoculation. [file Data_Sheet_1.zip › Figure 2 original images of gels/B.TIF]

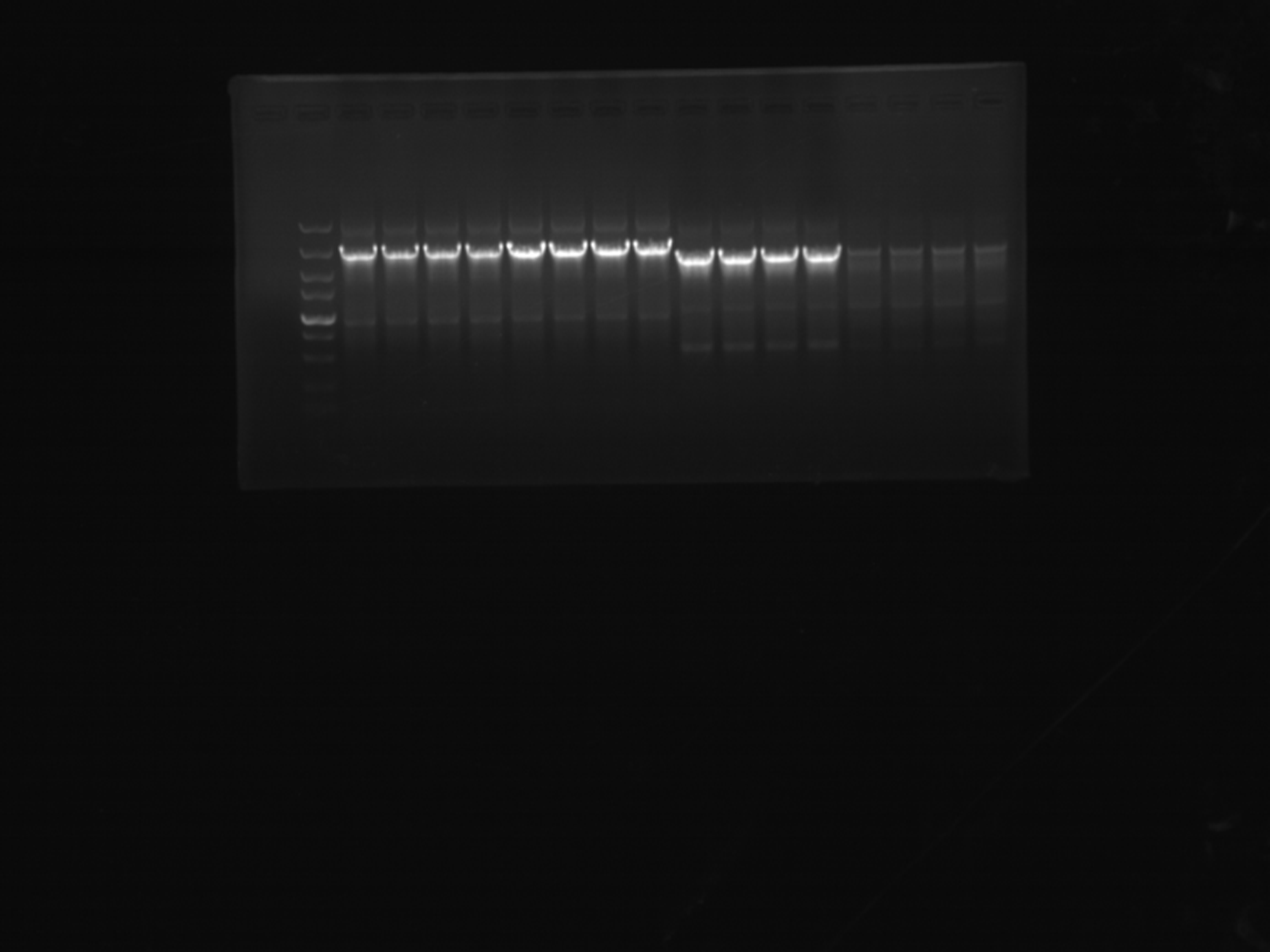

Supplement: Supplementary Figure 1 — Microscopic observation of mycelial structure of S. scitamineum. Photographs were taken 42 h after inoculation. [file Data_Sheet_1.zip › Figure 2 original images of gels/C.TIF]

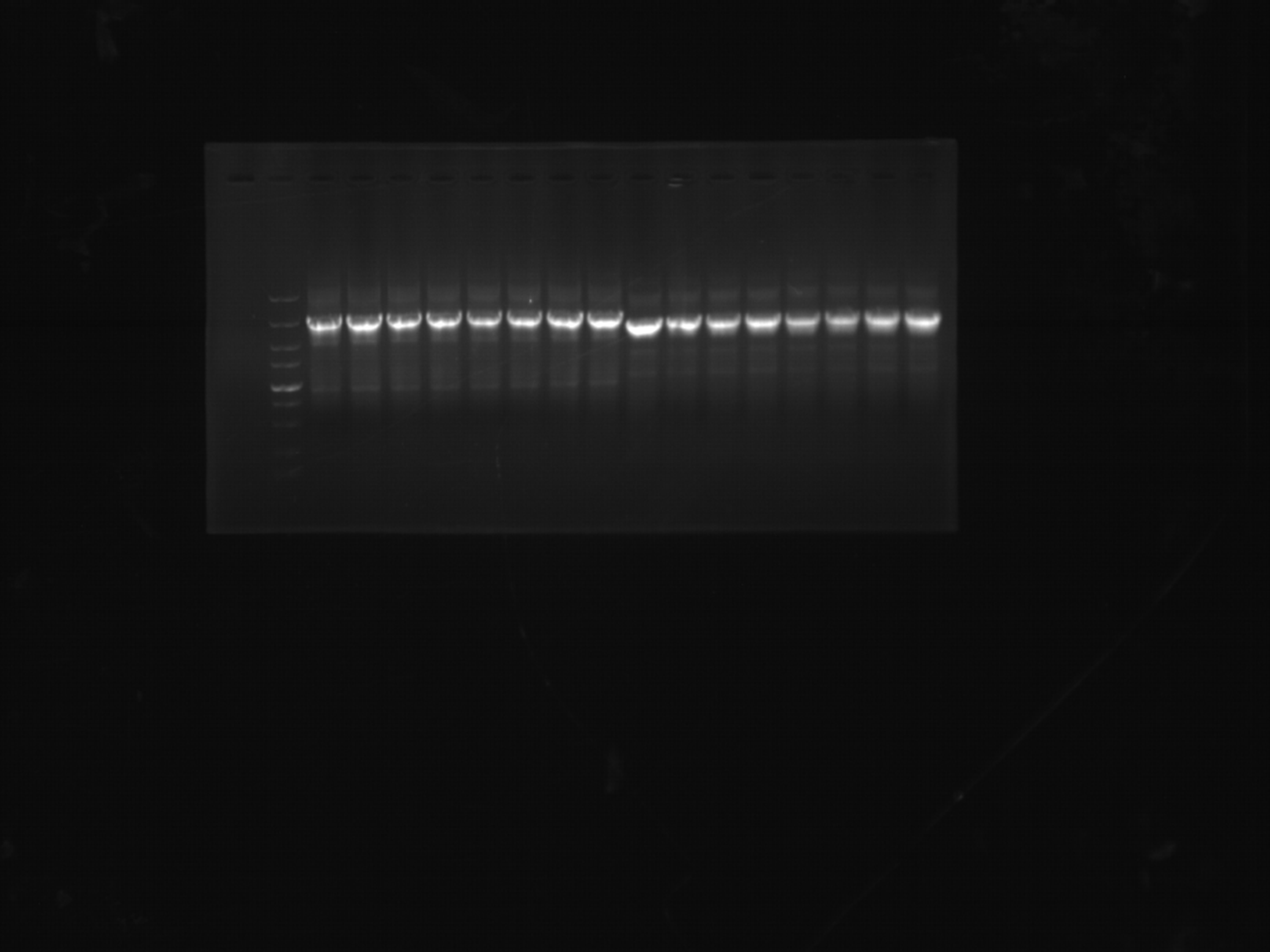

Supplement: Supplementary Figure 1 — Microscopic observation of mycelial structure of S. scitamineum. Photographs were taken 42 h after inoculation. [file Data_Sheet_1.zip › Figure 2 original images of gels/D.TIF]

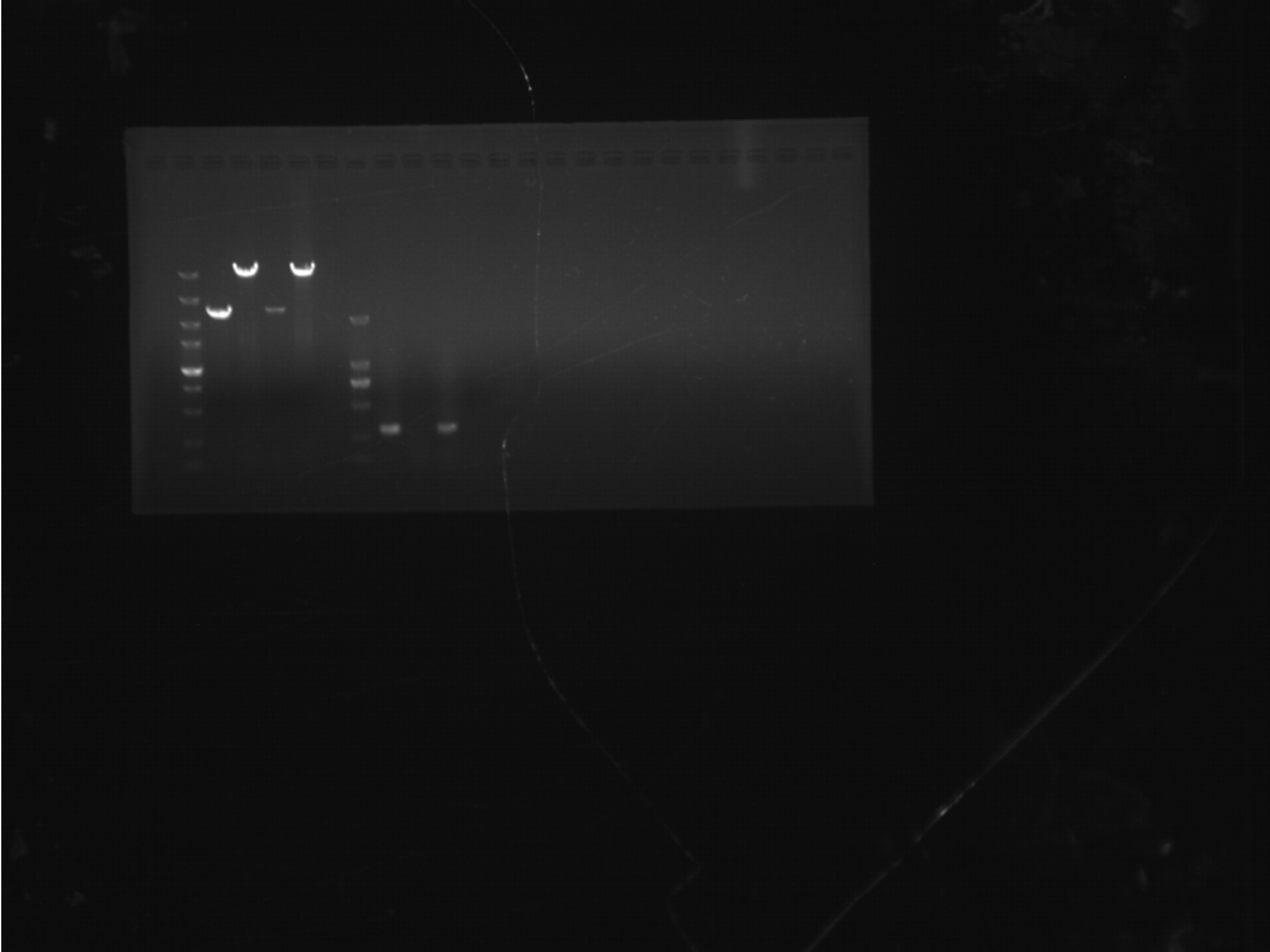

Supplement: Supplementary Figure 1 — Microscopic observation of mycelial structure of S. scitamineum. Photographs were taken 42 h after inoculation. [file Data_Sheet_1.zip › Figure 2 original images of gels/E.TIF]

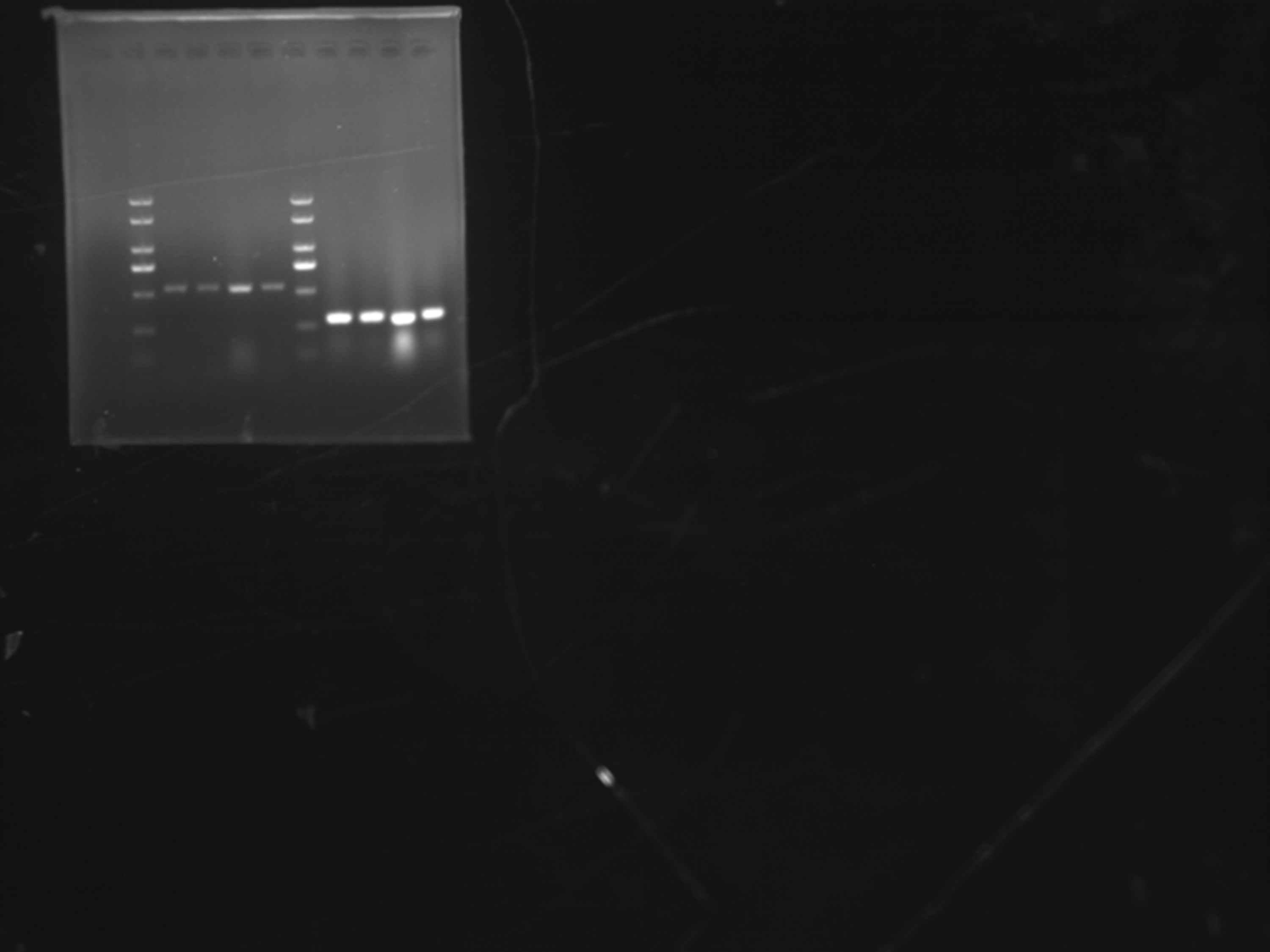

Supplement: Supplementary Figure 1 — Microscopic observation of mycelial structure of S. scitamineum. Photographs were taken 42 h after inoculation. [file Data_Sheet_1.zip › Figure 2 original images of gels/F.TIF]

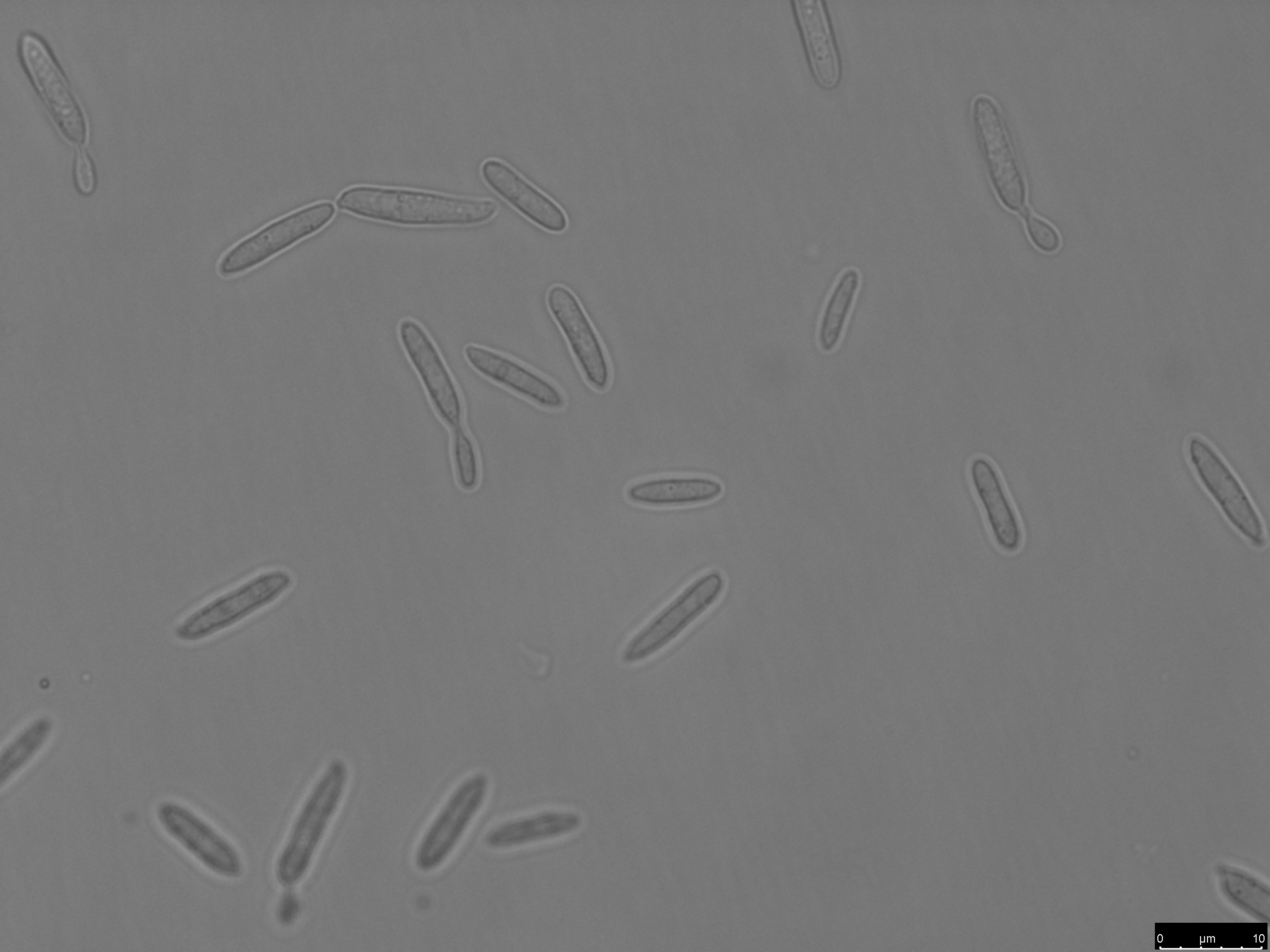

Supplement: Supplementary file 2 [file Data_Sheet_2.zip › Figure 5 morphology/1.tif]

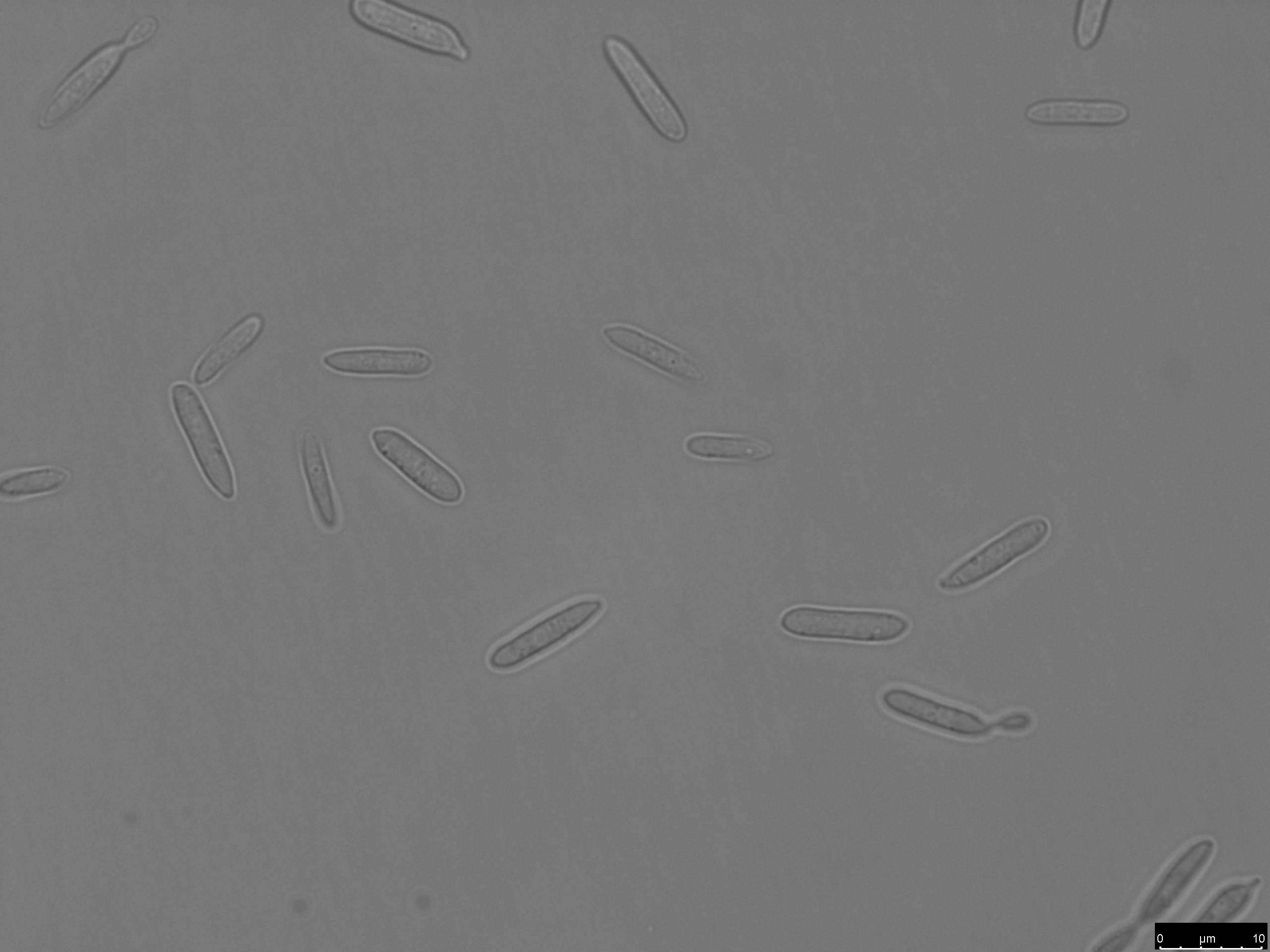

Supplement: Supplementary file 2 [file Data_Sheet_2.zip › Figure 5 morphology/2.tif]

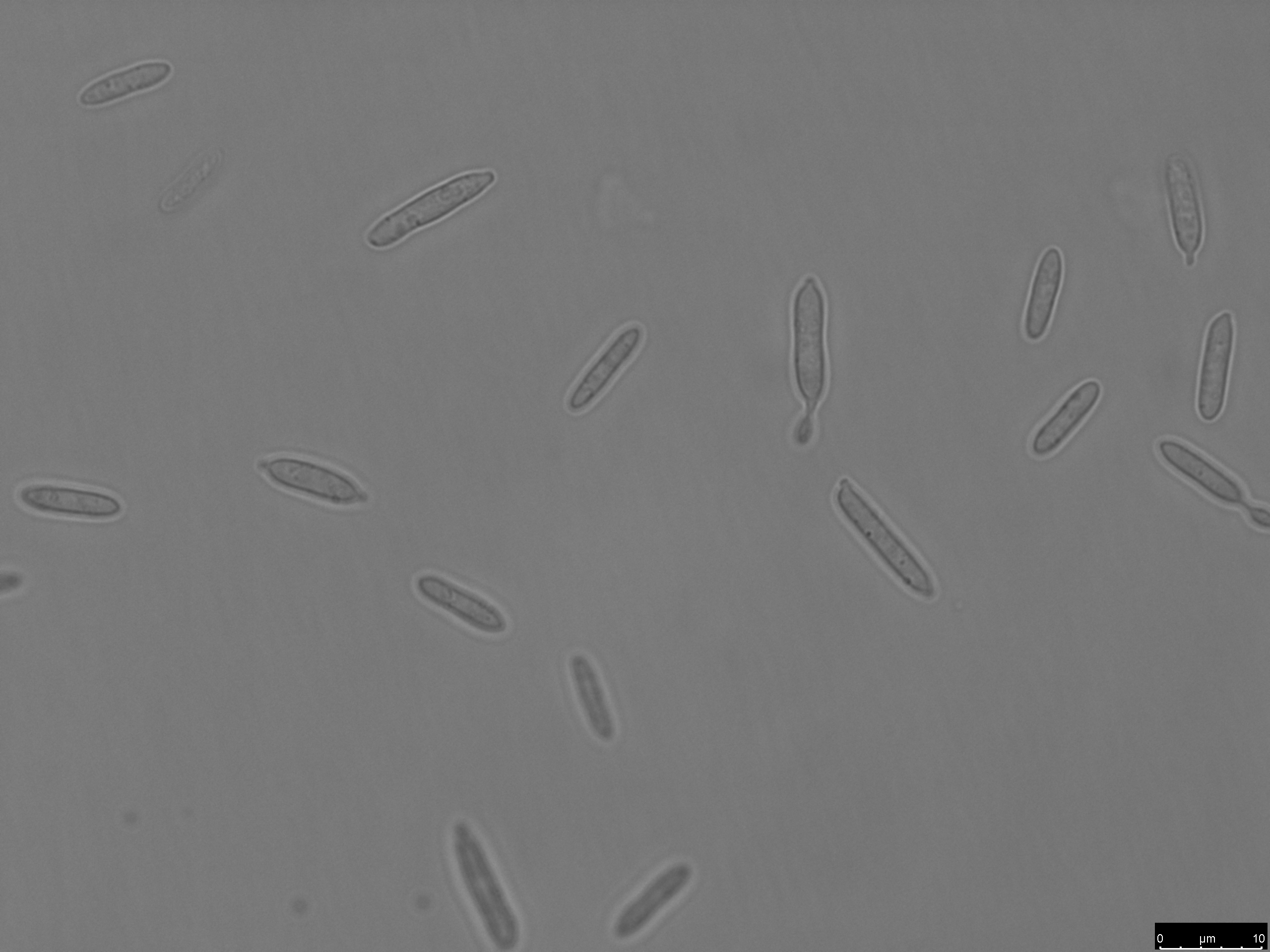

Supplement: Supplementary file 2 [file Data_Sheet_2.zip › Figure 5 morphology/3.tif]

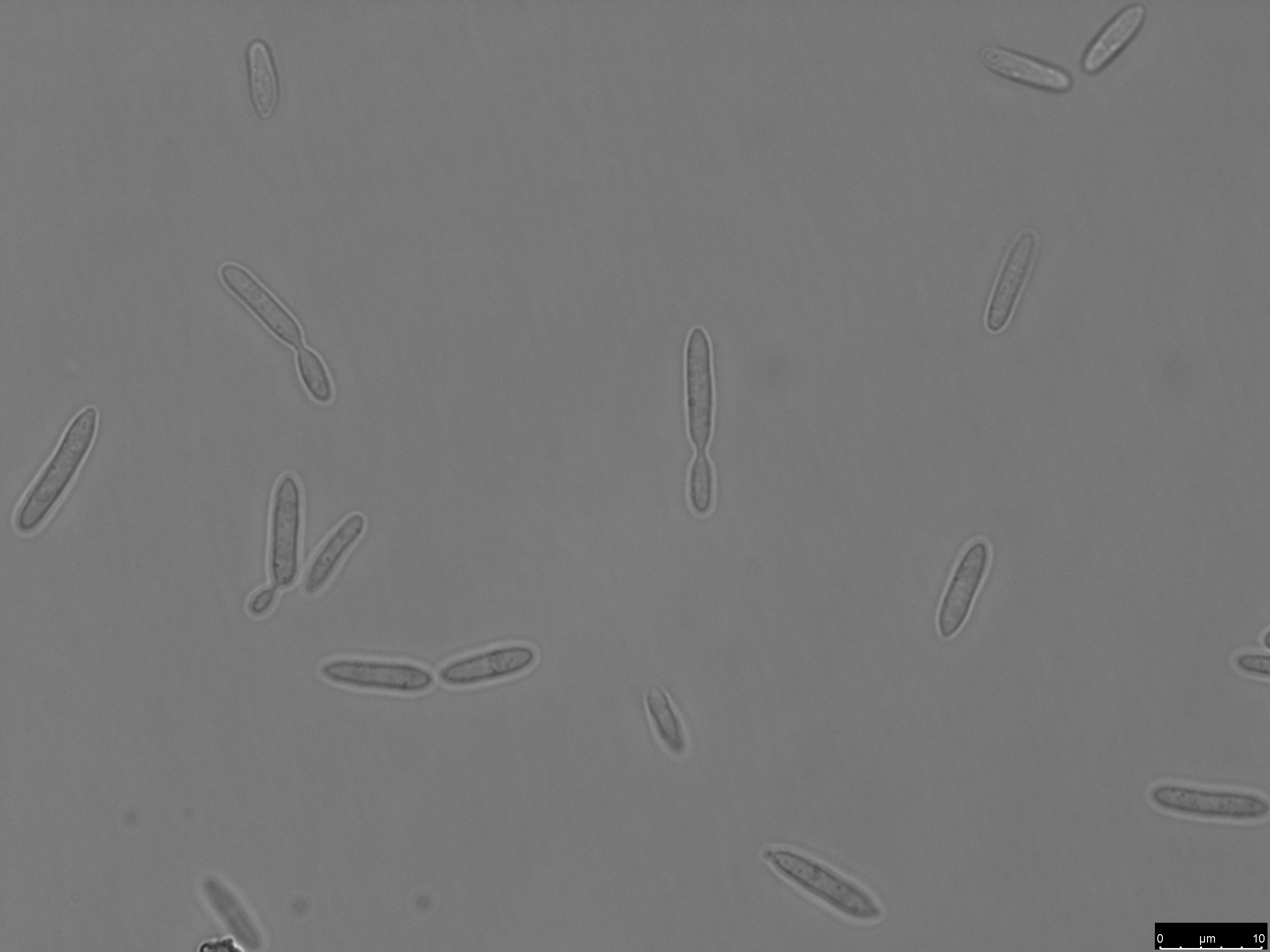

Supplement: Supplementary file 2 [file Data_Sheet_2.zip › Figure 5 morphology/4.tif]

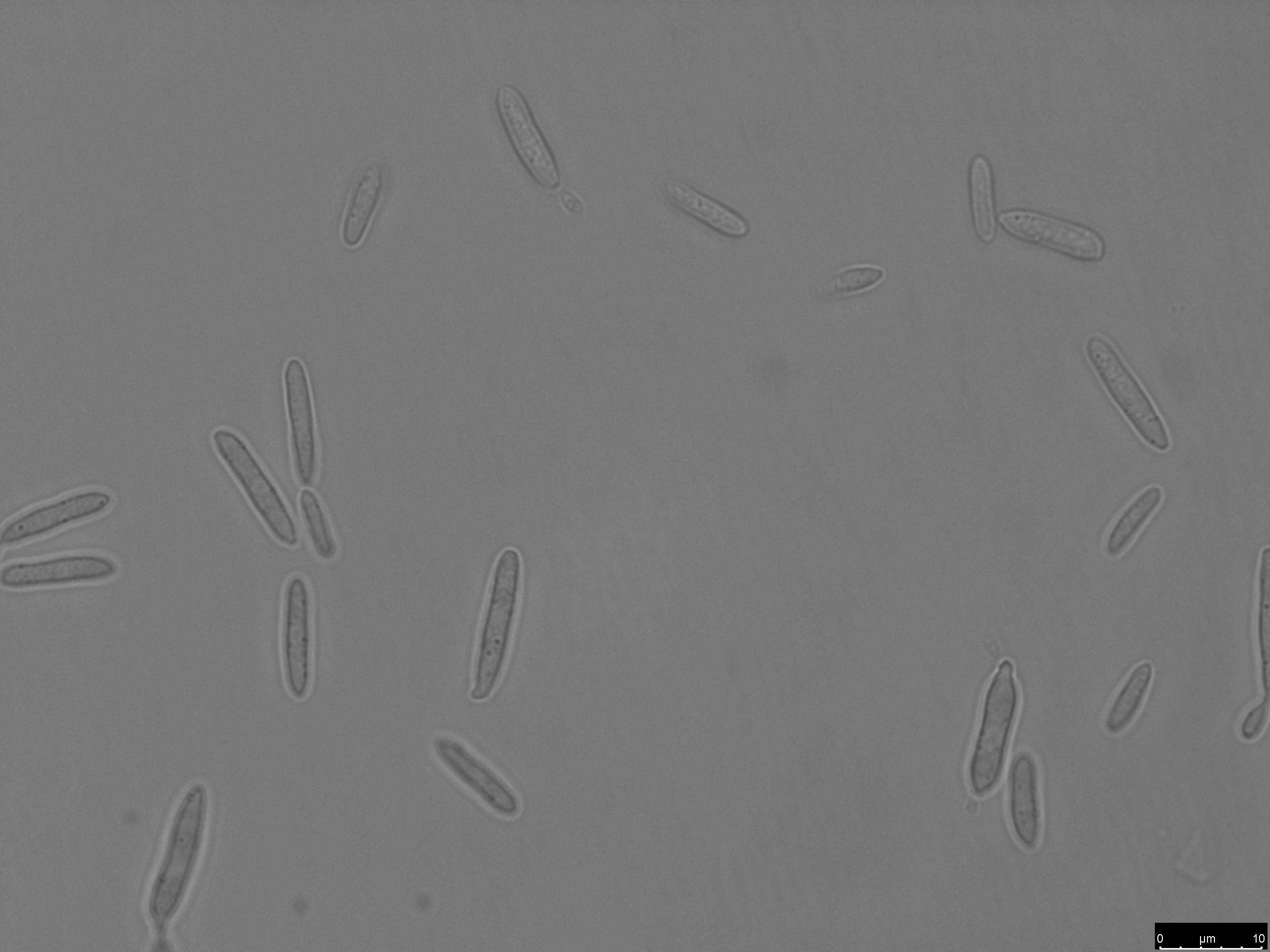

Supplement: Supplementary file 2 [file Data_Sheet_2.zip › Figure 5 morphology/5.tif]

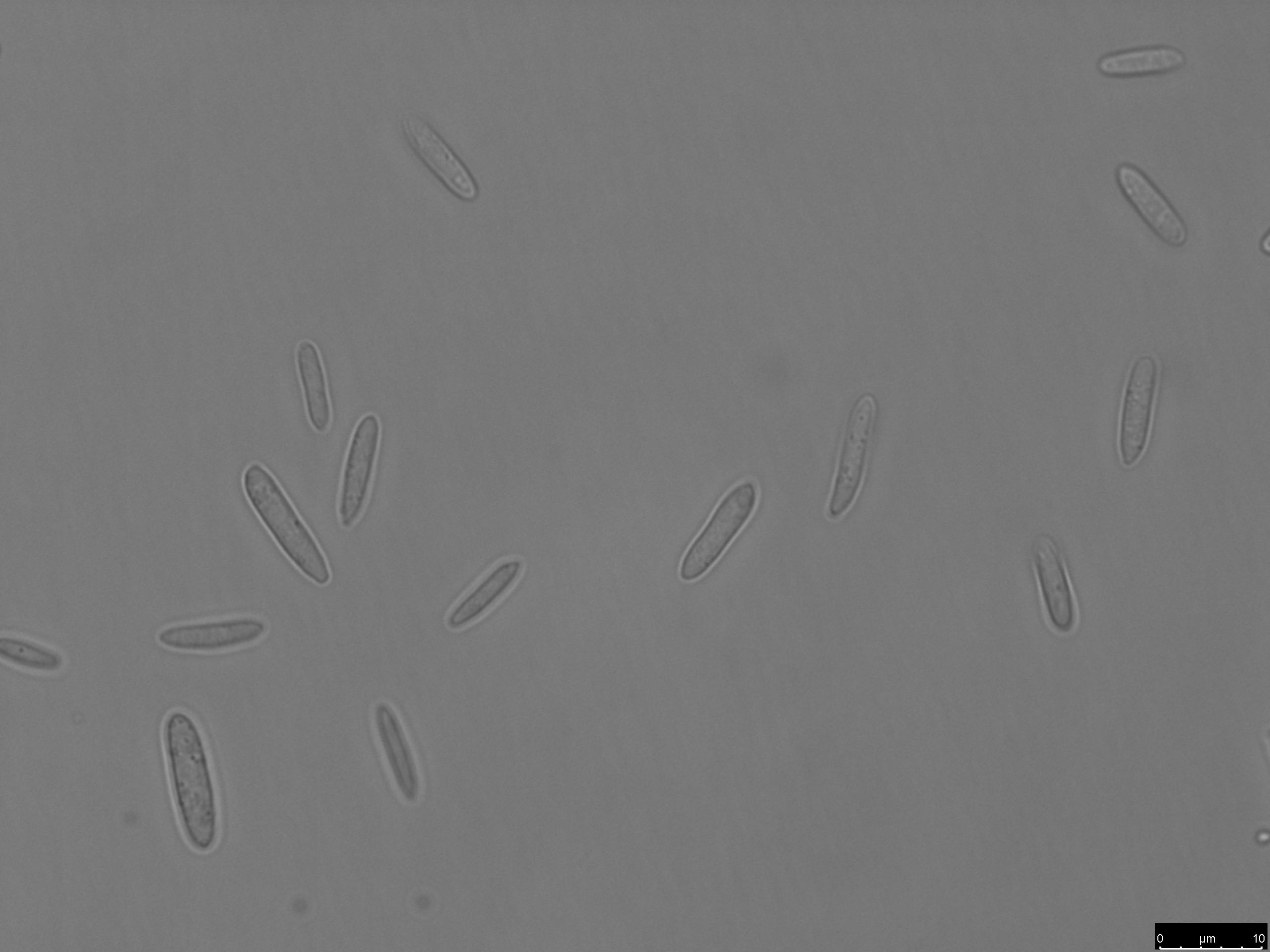

Supplement: Supplementary file 2 [file Data_Sheet_2.zip › Figure 5 morphology/6.tif]

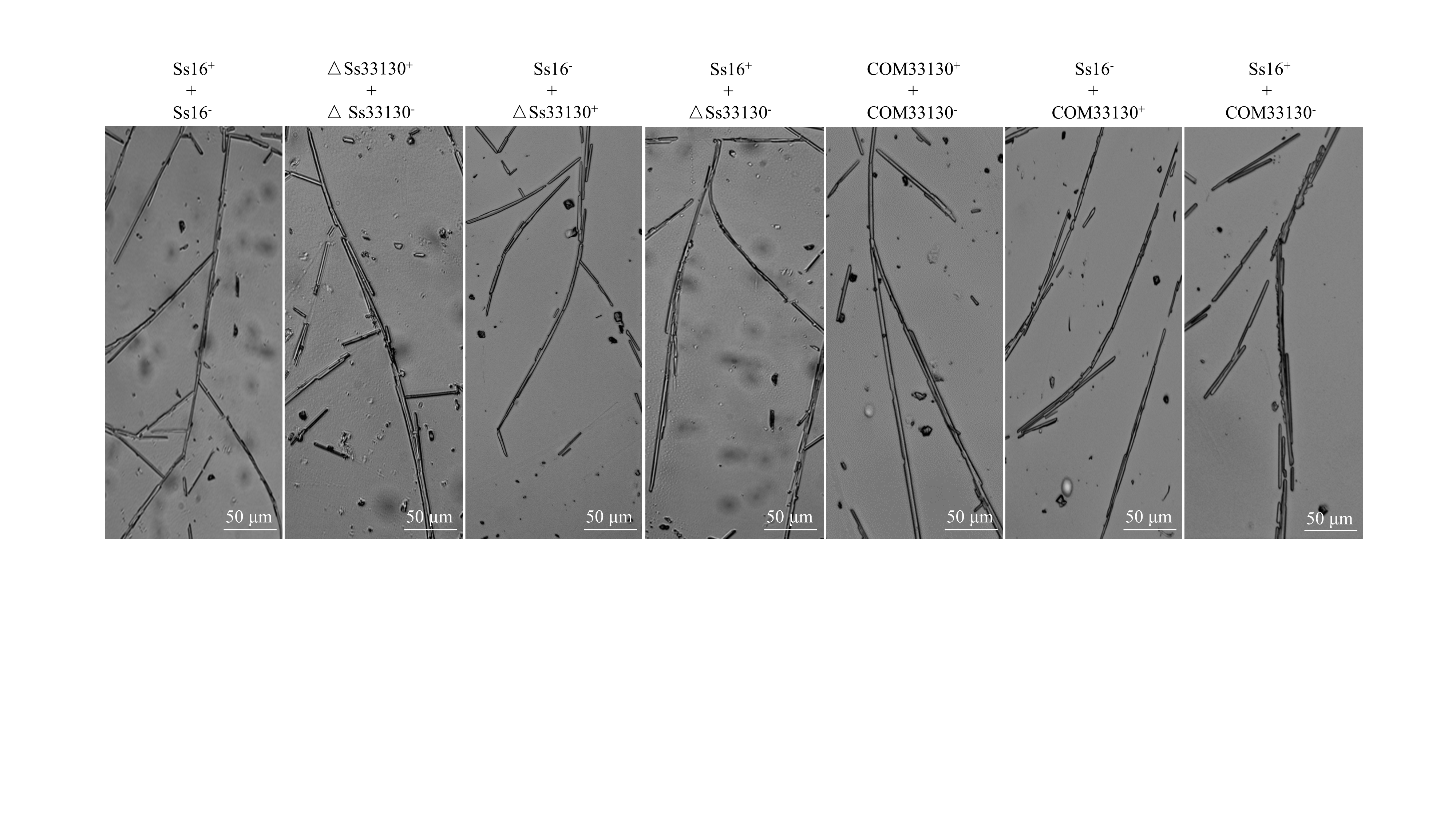

Supplement: Supplementary file 3 [file Image_1.TIF]
